# Zebrafish in Translational Cancer Research: Insight into Leukemia, Melanoma, Glioma and Endocrine Tumor Biology

**DOI:** 10.3390/genes8090236

**Published:** 2017-09-20

**Authors:** Aurora Irene Idilli, Francesca Precazzini, Maria Caterina Mione, Viviana Anelli

**Affiliations:** Laboratory of Experimental Cancer Biology, Cibio, University of Trento, Via Sommarive 9, 38123 Trento, Italy; aurorairene.idilli@unitn.it (A.I.I.); f.precazzini@unitn.it (F.P.); mariacaterina.mione@unitn.it (M.C.M.)

**Keywords:** zebrafish, cancers, cancer models, translational research, chemical-genetic screens, transplantation, melanoma, leukemia, glioblastoma, endocrine tumors

## Abstract

Over the past 15 years, zebrafish have emerged as a powerful tool for studying human cancers. Transgenic techniques have been employed to model different types of tumors, including leukemia, melanoma, glioblastoma and endocrine tumors. These models present histopathological and molecular conservation with their human cancer counterparts and have been fundamental for understanding mechanisms of tumor initiation and progression. Moreover, xenotransplantation of human cancer cells in embryos or adult zebrafish offers the advantage of studying the behavior of human cancer cells in a live organism. Chemical-genetic screens using zebrafish embryos have uncovered novel druggable pathways and new therapeutic strategies, some of which are now tested in clinical trials. In this review, we will report on recent advances in using zebrafish as a model in cancer studies—with specific focus on four cancer types—where zebrafish has contributed to novel discoveries or approaches to novel therapies.

## 1. Introduction

In the last 15 years zebrafish has emerged as a powerful model for studying both developmental processes and human diseases. Using zebrafish in cancer research provides several advantages including external fertilization, rapid development, high fertility and small size of the adult animal. Moreover, the major organs are fully developed by 5 days post fertilization (dpf). Embryos and larvae are transparent, so that the development of cancer at early stages can be easily followed under a microscope. The generation of stable transgenic lines is fast (6 months) and the availability of fluorescent lines marking organs leads to the observation of biological and disease processes in real time [1]. At the level of genome conservation, 71.4% of human genes have at least one zebrafish orthologue [2]. Besides these positive aspects, there are a few limitations including the presence of many duplicate genes, that complicates genetic manipulation, and the temperature they need to be raised to (28 °C), which is below the optimal temperature for growth and survival of mammalian cells (37 °C). Moreover, many commercially available antibodies do not recognize zebrafish proteins, making proteomic studies difficult. Despite these limitations, in the last decade zebrafish has been used in cancer research to model several types of cancer using different methods, including treatment with carcinogens, transgenesis, and transplantation of zebrafish or mammalian tumor cells [3]. 

Transgenic technology enables the formation of specific types of tumor by the expression of human oncogenes under tissue specific promoters. On the other hand, xenotransplantation of mammalian tumor cells into zebrafish larvae provides a novel way of studying human tumor growth in vivo and to monitor, at the same time, tumor angiogenesis and inflammation [4]. Finally, the large clutches of offspring and the low maintenance costs make this model suitable for large-scale chemical screens [5], to simultaneously assess drug efficacy and toxicity in vivo, bringing the identified hits more rapidly into the clinic.

In this review, we provide an overview of four different cancers (leukemia, melanoma glioblastoma and endocrine tumors) that have been modeled in zebrafish, highlighting key advances in understanding their biology. However, these models represent only a few zebrafish cancer models studied so far, as several other models have been generated and extensively characterized, including neuroblastoma [6] and pancreatic cancer [7]. We will discuss the potentials of translating discoveries made in the zebrafish system into preclinical and clinical investigations with the aim of identifying new therapeutic approaches for cancer.

## 2. Zebrafish as a Tool to Study Human Leukemia

The high conservation between human and zebrafish hematopoiesis [8] has stimulated the development of zebrafish models of hematopoietic malignancies to elucidate the molecular pathogenesis of these malignancies and expedite the preclinical investigation of novel therapies [9].

### 2.1. Zebrafish Hematopoietic Program

Hematopoiesis in zebrafish occurs in two successive waves—primitive and definitive [10]. The primitive wave originates in the intermediate cell mass (ICM) and give rise only to cells of the erythroid and myeloid lineage. The definitive wave produces hematopoietic stem cells (HSCs) capable of generating and repopulating the entire hematopoietic system. Starting from 26 h post fertilization (hpf) definitive HSCs emerge from hemogenic endothelial cells of the dorsal aorta in the aorta-gonad-mesonephros (AMG) region. Then HSCs migrate and seed the caudal hematopoietic tissue at 48 hpf, which is an expansion of the posterior blood island (PBI) and acts as transient hematopoietic site that give rise to erythrocytes, myeloid cells and platelets.

HSCs from the AGM region colonize the kidney around 48 hpf. Kidney marrow, which is functionally similar to mammalian bone marrow, gives rise to all blood lineages, including erythroid, myeloid, thromboid and lymphoid cells for the larval and adult zebrafish [10]. At around 54 hpf, common lymphoid progenitor (CLP) cells from the AGM region seed the thymus, which is the site for maturation of lymphoid T cells. Despite differences in the physiological location of the hematopoietic niche, the genetic programs utilized in each tissue are well conserved across species [10,11].

### 2.2. Human Leukemias

Leukemias are hematopoietic malignancies that can present themselves with a variety of different features. The most common types of leukemias occurring in humans and modeled in zebrafish belong to four major types, classified according to cell type and rate of growth: acute lymphoblastic leukemia (ALL), chronic lymphocytic leukemia (CLL), acute myeloid leukemia (AML) and chronic myeloid leukemia (CML) [12]. ALL, the most common childhood cancer and an important cause of morbidity among haematological malignancies in adults [13] can be divided into 2 classes, B-ALL and T-ALL, depending on the progenitors they are arising from (B- or T-cells) [14]. Over the past several years, genetic profiling—including microarray analysis and genome sequencing—have helped with identifying multiple key cellular pathways that are frequently mutated in leukemias [14,15,16,17]. This is now providing opportunities for personalized approaches to treatment that are based on individual mutational profiles. These mutations could be easily recapitulated in zebrafish using Crispr/Cas9 technology [18]. Multiple genetic changes, including chromosomal translocations, mutations and deletion led to leukemogenic transformation [19]. In the following Sections, we will discuss which models of leukemia have been generated so far in zebrafish, and how they have contributed to the understanding of the biology of leukemia’s initiation and progression.

### 2.3. Zebrafish Transgenic Models of T-Cell Acute Lymphoblastic Leukemia (T-ALL)

Acute lymphoblastic leukemia is due to malignant transformation and proliferation of lymphoid progenitor cells in the bone marrow, blood and extramedullary sites. T-ALL represents 10–15% of pediatric and 20–25% of adult cases of ALL in Europe, US and Japan [20,21]. Despite a high rate of response to chemotherapy, only 30–40% of adult patients with ALL will achieve long term remission. Treatments options in adult ALL have been adapted from pediatric protocols. Unfortunately, while 90% of pediatric ALL achieve long-term survival, the survival rate is not so high in adults [13]. Standard chemotherapy treatments include vincristine, corticosteroids and anthracycline [22,23]. Current research efforts are dedicated to developing targeted therapies to limit chemotherapy—which causes devastating side effects—and to increase treatment efficacy. However, development of targeted therapies requires the knowledge of the signaling pathways involved in cancer progression. To this aim, several zebrafish leukemia models have been generated and used, leading to insight in T-ALL pathogenesis and providing pathway-specific tools for chemical screens [24,25].

The first T-ALL zebrafish transgenic model was generated in 2003 by Look and colleagues by expressing mouse *c-Myc* fused to enhanced green fluorescent protein (E*GFP)* (*EGFP-mMyc*) under the control of the zebrafish *rag2* promoter [24]. The beauty of this model was to make it possible to monitor for the first time the dissemination of EGFP-labeled leukemic cells under a fluorescent microscope. However, as the onset of leukemia is very rapid in this fish (30 days of age), it could only be maintained through in vitro fertilization. To overcome this problem the same group generated a conditional transgene in which the *EGFP-mMyc* oncogene is preceded by a loxed *dsRED2* gene and the expression of *Myc* is controlled by Cre-mediated recombination of the *LoxP-dsRED2-loxP* cassette upon *Cre* mRNA injection [26]. This fish has red fluorescent thymocytes and does not develop leukemia when not injected with *Cre* mRNA recombinase. 

Transgenic progeny can be induced to develop T-ALL by injecting *Cre* mRNA into one cell stage embryos. These T-ALL zebrafish co-express the transcription factors *scl* and *lmo2*, crucial for development of all blood lineages, and resemble the most common and most treatment-resistant molecular subtype of T-ALL in human. A further improvement of this model was the generation of a transgenic line where *Cre* expression was controlled by a heat-shock promoter, *Hsp70* [27]. After heat-shock treatment at the larval stage, 81% of double-transgenic fish developed T-lymphoblastic lymphoma, which rapidly progressed to T-ALL. A second conditional zebrafish model of T-ALL, not based on the Cre/lox system but induced by tamoxifen was generated in the laboratory of Look [28]. After induction with 4-Hydroxytamoxifen (4HT), MYC was activated and fish developed T-ALL leukemia. Interestingly, withdrawal of 4HT results in T-lymphocyte apoptosis and tumor regression. Thanks to this transgenic model the authors discovered that loss-of-function mutations in the two zebrafish *pten* genes, or the expression of constitutively active AKT2, render tumors independent of the *MYC* oncogene. A second observation was that MYC reduces *pten* mRNA levels, suggesting that protein kinase B (AKT) pathway activation downstream of MYC is responsible of tumor progression [28].

Another pathway often activated in T-ALL is the NOTCH pathway. Activation of NOTCH1 contributes to the pathogenesis of over 60% of T-ALL [29,30]. Griffin and colleagues [31] developed a human NOTCH1-induced T-cell leukemia zebrafish model, where the intracellular portion of NOTCH1 (ICN1) is express under the *rag2* promoter. Forty four percent of injected fish developed a T-cell lymphoproliferative disease at about 5 months that invaded several tissues throughout the fish causing an aggressive and lethal leukemia when transplanted into irradiated recipient fish. Moreover, leukemia onset was dramatically accelerated when this transgenic line was crossed with another line overexpressing the zebrafish *bcl2* gene, indicating synergy between the NOTCH pathway and the BCL2-mediated antiapoptotic pathway. Thus, this fish line could be used in genetic modifier screens to reveal other genes that cooperate with NOTCH1 to induce T-ALL.

All these transgenic models have led to important insights into the pathogenesis of the disease and have been used in chemical screens and transplantation experiments aiming at a better understanding of T-ALL biology. In the next Sections, we will explore in detail how zebrafish genetic and transplantation models of acute leukemia have been used to provide an unprecedented opportunity to conduct rapid, phenotype-based screen to understand T-ALL biology and find new strategies to block its recurrence.

### 2.4. T-ALL Transplantation in Zebrafish

To dissect the mechanisms underlying self-renewal and the drug resistance of leukemia-propagating cells (LPCs), zebrafish leukemia cells have been transplanted from one zebrafish to other—whether genetically different or not—zebrafish (allotransplantation). The first transplantation experiment was performed in the Look’s laboratory after the generation of the first zebrafish T-ALL transgenic model [24]. Intraperitoneal transplantation of zebrafish GFP-positive leukemic cells in irradiated wild type adult zebrafish led them to study the dissemination of leukemic cells. These cells begun to spread through the peritoneal cavity within 14 days after injection, homing in the thymus between 14 and 26 days after injection. Moreover, all the injected fish had leukemic cells infiltrating the region adjacent to the olfactory bulb, suggesting that this region is a preferred site for the homing of immature T-cells. Next, to quantify LPCs and study their biology, Langenau and colleagues generated and transplanted *MYC*-induced T-cell acute lymphoblastic leukemia in the CG1 strain, a syngeneic fish generated by in vitro fertilization of eggs from a Golden strain female with ultraviolet-inactivated sperm and subjected to heat shock to produce gynogenetic diploid animals [32]. Transplantation in this strain does not need irradiation to immuno suppress, thus overcoming problems related to the irradiation in recipient adult zebrafish, such as mortality or regression of the tumor due to the re-establishing of immunocompetent cells. These studies revealed that self-renewing leukemic cells are abundant in T-ALL and comprise between 0.1% and 15.9% of the T-ALL mass. An important discovery that came out of these studies is that T-ALL can be initiated from a single cell and that different leukemic clones exhibit wide differences in tumor-initiating potential. The authors also set up a high-throughput imaging method based on an LED-fluorescent microscope that allowed the simultaneous imaging of large numbers of fluorescent transgenic animals, facilitating the rapid screening of engrafted animals [32].

Using a mosaic transgenic T-ALL model, Langenau and colleagues [33] found that NOTCH expression induced a significant expansion of pre-leukemic clones. However, a majority of these clones were not fully transformed and could not induce leukemia when transplanted into recipient animals. Limiting-dilution cell transplantation revealed that NOTCH signaling does not increase the overall frequency of LPCs, either alone or in collaboration with *MYC*, indicating that a primary role of NOTCH signaling in T-ALL is to expand a population of pre-malignant thymocytes. Then, a subset of them acquires further mutations to become transformed LPCs [33].

To study the mechanisms by which clonal evolution and intratumoral heterogeneity drive cancer progression, the functional differences between single T-ALL clones were assessed using a zebrafish transgenic model [34]. To this aim, a large-scale cell transplantation based screen was performed in which syngeneic zebrafish were engrafted with single, fluorescently labeled clones isolated from primary *MYC*-induced T-ALL. This approach mimics the process by which a single cell can re-initiate leukemia and induce relapse. The results of this screen revealed functional variation within individual clones, with a minority of clones enhancing growth rate and leukemia-propagating potential with time. One important discovery in this study is that activation of the AKT pathway was acquired in a subset of the clones, and increased the number of leukemia-propagating cells through activating mTORC1 and elevated growth rate, probably by stabilizing MYC. Moreover, the AKT inhibitor MK2206 can sensitize refractory T-ALL cells to dexamethasone-induced killing in vivo. These results demonstrate that T-ALL clones can spontaneously develop resistance to chemotherapy as a result of clonal evolution and that this can occur without selection induced by prior drug exposure [34].

An important contribution for optimized cell transplantation and direct visualization of fluorescently labelled cancer cells at single-cell resolution using zebrafish was made by Langenau and colleagues [35], who developed the first optically clear immune-compromised *rag2^E450fs^* (casper) zebrafish. This fish was generated by zinc finger nuclease mutation of the plant homeodomain (PHD) domain of recombination activating gene 2 (*rag2*) gene. Mutations in this domain disrupt RAG2 functions; mutations in the same domain are found in patients with Omenn syndrome [36], an autosomal recessive severe combined immunodeficiency (SCID) characterized by impaired T and B cell receptor rearrangement and reduced number of functionally mature lymphocytes in human patients [36]. Primary *MYC*-induced T-ALL cells were transplanted into the peritoneal cavity of recipient animals. Homozygous *rag2^E450fs^* mutant fish robustly engrafted T-ALL by 30 days post transplantation, even in the absence of prior immunosuppression by gamma-irradiation. By contrast, heterozygous and wild type siblings failed to engraft T-ALL. 

Although the *rag2^E450fs^* model is an important advance in zebrafish transplant technology, the model is not optimal, as the homozygous fish do not breed. Moreover, the mutation is hypomorphic and the fish lack T cells, but have variable B cell defects that differ greatly between fish, likely impacting engraftment potential within individual animals. To overcome these limitations, the same laboratory [37] generated new mutant lines by transcription activator-like effector nucleases (TALEN): (1) a DNA-dependent protein kinase mutant (*prkdc^D3612fs^*) and (2) a janus kinase 3 mutant (*jak3^P369fs^*). Both mutants have indels causing frameshifts and premature stop codons resulting in truncated protein products. Extensive characterization of these mutants showed that *prkdc* deficiency results in loss of mature T and B cells and *jak3* mutants lack T and putative Natural Killer cells. Although all mutant lines engraft fluorescently labeled normal and malignant cells, only the *prkdc* mutant fish can be bred as homozygotes and survive well after cell transplantation [37]. To study the stochastic events governing the emergence of underrepresented clones within primary leukemia, the authors transplanted equal numbers of T-ALL cells with similar growth kinetics, labeled with 3 different fluorophores, in *prkdc* mutant casper line. They found that, 26 days after transplantation, leukemias were dominated by a single fluorescent clone. The type of dominant clone was stochastically determined, thus supporting a model where clonal dominance can emerge as consequence of neutral stochastic drifts. These results show how these models could be important to dynamically visualize processes of clonal dominance and cancer progression at single-cell resolution in engrafted immunosuppressed transparent fish [37].

### 2.5. Chemical Screen to Find New Pathways and Therapies in T-ALL

A chemical screen to find new pathways modulating definitive HSCs during zebrafish embryogenesis was performed by the Zon laboratory in 2007 [38] using in situ hybridization for *runx1* and *cmyb*, two transcription factors required for mammalian HCS formation as read out. Wild type embryos were incubated with a panel of biological active compounds at the stage of 3-somites and analyzed at 36 hpf. This study identified prostaglandin E2 (PGE2) as a regulator of HSCs number. This result has been confirmed by in vitro and in vivo preclinical studies in the murine model to evaluate the therapeutic potential of 16,16-dimethyl PGE2 (dmPGE2, a PGE2 form resistant to metabolism with a prolonged half-life in vivo), in umbilical cord blood (UCB) transplantation [39]. A phase I study to examine the safety of ex vivo dmPGE2 treatment to expand UCB HSC for transplantation in human patients has been completed (Identifier: NCT01527838 at clinicaltrial.gov). Results from this trial demonstrated clearly safety with durable engraftment of dmPGE2-treated UCB. In particular, neutrophil engraftment was observed in 100% of patients and dmPGE2-treated UCB displayed enhanced engraftment by outcompeting the untreated UCB unit in 10 out of 12 patients. Moreover, the time of the engraftment was shorter in dmPGE2-treated UCB compared to the control [40]. These studies are very important advances in the treatment of leukemia patients as ex vivo expansion of HSCs prior to stem cell transplantation may improve reconstitution of hematopoiesis and immune functions in patients.

Following this first screen using wild type fish, other screens have been performed to study misregulated pathways in T-ALL using zebrafish transgenic lines. However, as zebrafish models for this disease showing an embryonic phenotype have not yet been generated, other strategies have been used. Starting from the assumption that immature T cells and leukemic T lymphoblasts share similar pathways or biological processes on which both depend, Trede and colleagues [41] performed a chemical screen using the transgenic line *Tg(lck:lck-EGFP)*, that expresses EGFP-fused *lck* under the control of the T-cell specific tyrosine kinase (*lck*) promoter [42]. After incubating this fluorescent line for 2 days in a 96 well plate with 26,400 compounds from the Chemibridge DIVERSet library, Lenaldekar (LDK) was found to eliminate immature T-cells in developing zebrafish without affecting the cell cycle in any other cell type. Moreover, adult fish with *c-MYC* induced T-ALL treated with LDK underwent long term remission. This drug is well tolerated in mice, has favorable pharmacokinetics, and slows the in vivo growth of human T-ALL in murine xenografts. Moreover, LDK is active against primary human leukemia cells, including BCR-ABL(T315I) mutated cells, therapy-refractory B-ALL, and CML samples [41]. After studying the mechanisms by which LDK eliminates immature T-cells, the authors found that LDK has two independent activities: (1) delaying mitosis and (2) inhibiting the phosphoinositide 3-kinase/AKT/mechanistic target of rapamycin (PI3K/AKT/mTOR) pathway. All these observations led to the hypothesis that LDK could represent a new, targeted approach to leukemia treatment [41].

In 2014, Aster and colleagues [43] performed two complementary screens to identify drugs approved by the Food and Drugs Administartion (FDA) with activity against T-ALL. A screen was done using a zebrafish transgenic line expressing *c-MYC* in thymocytes [24] using 4880 FDA-approved drugs from 4 different libraries (Lopac, ICCB, Prestwick and Spectrum collection) and a parallel screen was done on KOPT-k1 cells, a NOTCH1-dependent T-ALL cell line. NOTCH1 is a crucial therapeutic target in T-ALL, but gamma secretase inhibitors (GSIs) used as a single agent in T-ALL preclinical model and in clinical trials showed modest effects, suggesting the need to combine NOTCH pathway inhibitors with other compounds. To this aim, an in vitro screen was performed to find small molecules that synergize with NOTCH inhibitors. Interestingly, the authors identified the antipsychotic drug perphenazine (PPZ) in both the in vivo and the in vitro screens. This drug was able to induce suppression of cell growth and apoptosis in fish, mouse, and human T-ALL cells through the activation of the tumor suppressor protein phosphatase 2A (PP2A). These results provide evidence that activation of PP2A in combination with NOTCH-targeting therapeutics might provide an effective T-ALL therapy [43].

As none of the transgenic T-ALL zebrafish models show an embryonic phenotype, xenografted leukemic cells in zebrafish embryos could be a pharmacologically relevant model for the screening of non-teratogenic drugs. Solary and colleagues [44] performed xenotransplantation experiments by injecting human leukemic cell lines and blast cells sorted from patients with acute myelogenous leukemia in 48 hpf larvae. After 1 day, larvae were treated with few different antileukemic drugs (Imatinib, oxaphorines, all-trans retinoic acid, 4EGI-1) for 2 days. The results showed that Imatinib and oxaphorines decreased the leukemic burden in xenografted animals, without affecting embryonic development.

### 2.6. Acute Myeloid Leukemia (AML) and Myelo-Erythroid Proliferative Disorder

AML involves the abnormal proliferation and differentiation of a clonal population of myeloid cells. Well-characterized chromosomal translocations, such as t(8;21) q(21;22), which leads to the expression of the *AML1-ETO* fusion gene [45], inv(8)(p11;q13), leading to the expression of the MYST3/NCOA2 fusion gene [46] and t(7;11)(p15;p15), leading to the expression of the chimeric fusion protein Nup98/HOXA9 [47] have been modeled in zebrafish.

Beside chromosomal rearrangements, molecular changes have also been implicated in the development of AML [48], including mutations in *Fl3*, *N/K-RAS*, *TP53*, *STAT3*, *NPMI* and *CEBPA*. Frequent mutations have been observed in genes involved in epigenetic regulation, encoding DNA methyltransferase 3A (*DNMT3A*), isocitrate dehydrogenase 1 (*IDH1*) and isocitrate dehydrogenase 2 (*IDH2*), as well as tet oncogene family member 2 gene (*TET2*) [49]. The presence of *DNMT3A*, *TET2*, *IDH1* or *IDH2* mutations may confer sensitivity to novel therapeutic approaches, including the use of demethylating agents. Zebrafish models carrying these mutations have not yet been generated.

The first model of AML was generated almost 10 years ago by Delva and colleagues [50] by expressing the *MYST3/NCOA2* fusion gene under the control of the *spi1 (pu.1)* promoter. After injecting the fusion gene at one cell stage, only 2 of 180 of injected fish developed AML, at 14 and 26 months of age respectively. Leukemia in these fish was characterized by an extensive invasion of kidneys by myeloid blast cells. The long latency and low incidence could indicate the necessity of additional genetic events for *MYST3/NCOA2* to induce AML.

In parallel with this first model, Peterson and colleagues [51] generated a transgenic zebrafish line that enables inducible expression (mediated by hsp70 promoter) of the human AML1-ETO oncogene, leading to AML already in embryos. Using this model, they discovered that AML1-ETO redirects myeloerythroid progenitor cells that are developmentally programmed to adopt the erythroid cell fate into the granulocytic cell fate. AML1-ETO activation by heat shock downregulates *scl* and *gata1* expression, 2 genes expressed in primitive erythroid cells. When the transgenic fish were treated with a histone deacetylase inhibitor, Trichostatin A, *scl* expression was restored and the accumulation of granulocytic cells caused by AML1-ETO was attenuated [51]. Given the fact that this inducible model shows an early embryonic phenotype, it was used in a large-scale chemical screen (described below) to identify suppressors of the in vivo effects of AML1-ETO.

In 2011, Berman and colleagues [52] generated an inducible Cre/lox transgenic zebrafish harboring human *NUP98-HOXA9* under the zebrafish *spi1 (pu.1)* promoter. Transgenic embryos had defects in early hematopoiesis, with an abundance of myeloid cells marked by *spi1*, loss of red cells marked by *gata1* and inhibition of terminal myeloid differentiation. Twenty-three percent of adult *NUP98-HOXA9*-transgenic fish developed a myeloproliferative neoplasm (MPN) at 19–23 months of age [52].

Following the expression of oncogenic RAS in endothelial cells, including the hemogenic endothelium of the dorsal aorta, Mione’s group observed the development of a myelo-erythroid proliferative disorder in transgenic fish [53]. The phenotype in larvae consisted of disrupted vascular system and expansion of the caudal hematopoietic tissue. An increased number of immature hematopoietic cells and arrest of myeloid maturation in kidney marrow was observed in few surviving juvenile transgenic fish. Two important observations of this study were that myeloid neoplasms could be generated by targeting the HSCs before their homing into the kidney marrow and that the abnormal hematopoietic phenotype was associated with a downregulation of the NOTCH pathway, suggesting that downregulation of this pathway is an important step in the pathogenesis of myelo-erythroid disorders [53].

### 2.7. Chemical Screen in Zebrafish AML Models

The AML1-ETO inducible transgenic model [51] was used to perform a chemical screen using a library of 2000 bioactive compounds with the aim to identify signaling pathways activated by AML1-ETO [25]. To this purpose, embryos were heat shocked for 60 minutes at 40 °C leading to downregulation of *gata1* expression. Ninety minutes after heat shock embryos were fixed and expression of *gata1* was evaluated by in situ hybridization. The results showed that nimesulide, a selective COX-2 inhibitor was able to antagonize the effect of AML1-ETO on hematopoietic differentiation. By studying the mechanisms by which nimesulide antagonizes the effects of the oncogene, they found that COX-2 signaling enhances beta-catenin expression leading to an expansion of hematopoietic progenitors [51]. These results might be important to find new therapies that specifically affect PGE2 signaling or inhibit beta-catenin dependent pathways. The results were further confirmed by experiments in mice, where AML1-ETO expression also induced COX-2 and activates beta-catenin in bone marrow cells [54]. Moreover, treating mice xenografted with leukemic cells SKNO-1 with nimesulide dramatically suppressed tumor formation and inhibited in vivo progression of human leukemia cells.

### 2.8. Summary and Translational Impact of Zebrafish Models in Leukemia Research

**Figure 1:**
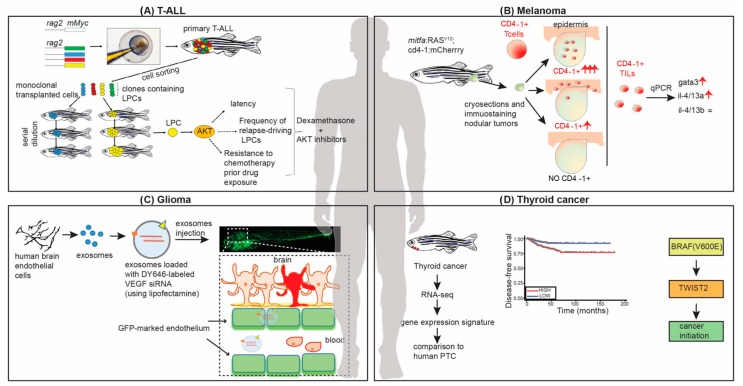
Novel aspects of tumor biology and translational approaches using zebrafish models. (**A**) Studying clonal evolution in T-cell acute lymphoblastic leukemia (T-ALL) using zebrafish: syngeneic zebrafish were engrafted with single, fluorescently labeled clones isolated from primary Myc-induced T-ALL. Individual clones show functional variation, with a minority of clones enhancing growth rate and leukemia-propagating potential. Protein kinase B (AKT) pathway was acquired in a subset of the clones, and increased the latency, the frequency of relapse due to the presence of leukemia-propagating cells (LPCs) and the resistance to chemotherapy in the absence of drug exposure. The study by Langenau and colleagues suggests that a combination of dexamethasone and AKT inhibitors could block tumor relapse [34]. (**B**) Studying the immunological phenotype of melanoma: nodular tumor from transgenic zebrafish were cryosectioned and analyzed by immunohistochemistry for CD4-1+ lymphocytes. Using this model Hurlstone and colleagues identified different lesions: tumor with extensive lymphocytes infiltration, little infiltration or no infiltration [55]. Quantitative (q) PCR analysis of CD4-1+ tumor infiltrating lymphocytes (TILs) reveals high levels of *gata3* and Th2-associated cytokine *il-4/13a* but not *il-4/13b*. This study suggests that tumor immuno responses can be faithfully studied in zebrafish models. (**C**) Studying new ways to deliver chemotherapeutics across the blood-brain barrier (BBB): exosomes were isolated from brain endothelial cells (bEND.3) and cultured in exosome-depleted FBS Media. Fluorescently labeled vascular endothelial growth factor (VEGF) small interfering RNA (siRNA) was loaded in exosomes by lipofectamine. Five days post fertilization (dpf) *Tg(fli1a:GFP)* zebrafish were injected with exosomes-siRNA into the common cardinal vein. Exosomes containing fluorescent siRNA crossed the BBB and were delivered in the brain, demonstrating that exosomes could be valid carriers of drugs across the BBB and could be studied in zebrafish [56]. (**D**) Using zebrafish to study endocrine tumors: zebrafish thyroid tumors harbor a gene expression signature that stratifies disease recurrence in patients with papillary thyroid cancer (PTC), identifying patients with significant lower risk of relapse. *Twist3*, the ortholog of the human *Twist2* was found upregulated in BRAF^V600E^-expressing thyrocytes. Loss of *twist3* expression using Crispr/Cas9 technology demonstrated the key role of this transcription factor in thyroid cancer initiation [57].

The generation of zebrafish transgenic models of leukemia led to major discoveries in the field, including a more detailed knowledge of signaling pathways governing leukemia progression and recurrence. Several evidences, from transgenic fish and transplantation experiments, highlight the role of the AKT pathway in T-ALL: functional mutations in *PTEN* genes or expression of constitutively active AKT2 render tumors independent from the MYC oncogene [28]. Transplantation experiments using serial dilution of leukemic zebrafish cells into immunocompromised or syngeneic recipient fish were pivotal to understand clonal evolution driving cancer progression. These experiments also led to the discovery that activation of the AKT signaling pathway is acquired stochastically in a subset of clones of untreated leukemias, and increases the number of leukemia propagating cells through the activation of mTORC1 [34]. The AKT pathway activation renders clonally evolved T-ALL cells insensitive to dexamethasone, a glucocorticoid used for the therapy of T-ALL. Clinical trials on T-ALL patients are ongoing to test the combination of dexamethasone and different AKT inhibitors, leading the way to further prospective use of zebrafish leukemia models in translational approaches (Figure 1A).

In the future, the availability of immunocompromised mutant fish will lead to the use of zebrafish in personalized medicine, where leukemic cells from patients could be transplanted in fish in order to study clonal evolution. Genome sequencing on the clones having growth advantage could reveal combinations of mutations that favor clonal expansion in one particular patient; drug combinations able to block the expansion could be tested in xenotransplanted fish. Drugs able to block clonal leukemia expansion in transplanted fish could then be used to prevent leukemia relapse in patients. The mutations found in human samples are already being recapitulated in zebrafish HSCs using Crispr/Cas9 editing technology, as reported at recent zebrafish meetings, and their effects in leukemia progression and recurrence will be investigated. Soon the results of these studies will contribute to the development of precision medicine in leukemic patients.

## 3. Zebrafish as a Model for Studying Human Melanoma

Melanoma is a malignancy of melanocytes, which are cells of neuroectodermal origin producing melanin [58]. They can be found throughout the body, mostly in teguments including not only the skin but also uvea, meninges and mucosal tissues [59]. The cutaneous form of melanoma is common in the Western world and causes the majority (75%) of deaths related to skin cancer [59]. The global incidence is 15–25 per 100,000 individuals. Sun exposure (ultraviolet, UV irradiation) is the major risk factor for cutaneous melanoma and leads to a genetic signature that is typical of melanoma [60]. Recent next generation sequencing studies have revealed that, with a median number of >10 mutations per megabase of DNA, melanomas have the highest mutational load of all human malignancies, represented by a large number of UV-signature mutations, predominantly C > T or G > T transitions, which are induced by UVB and UVA, respectively [61]. On the other hand, several studies suggest that melanoma is not exclusively UV-dependent. Melanomas can develop in non-sun-exposed skin or in internal organs. Nowadays, the mostly relevant molecular pathway for oncogenic and therapeutic purposes is the mitogen-activated protein kinase (MAPK) cascade. Common mutations include *BRAF^V600E^*, *NRAS^Q61L^* or *NRAS^Q61R^* and *HRAS* and *KRAS* mutations for cutaneous melanoma [62], *KIT^V559A^* for mucosal and acral melanoma and *GNAQ^Q209L^* or *GNA11^Q209L^* in uveal melanomas. Surprisingly, none of these mutations carries the typical UV signature, C > T or G > T transitions. The absence of typical UV signatures, however, does not completely exclude a causal role for UV radiation in the generation of these mutations [63].

### 3.1. Zebrafish Melanoma Models

The possibility to express human melanoma oncogenes in the melanocyte lineage allowed the generation of different melanoma models in zebrafish. An overview of the models with their driver mutations is described below.

#### 3.1.1. BRAF^V600E^/p53

The first zebrafish model for human BRAF^V600E^ under the melanocyte-specific promoter *mitfa* was generated in 2005 by Patton and Zon [64]. Mosaic BRAF^V600E^ expression generated large melanocytic spots on the adult zebrafish that were similar to nevi. The transgenic zebrafish line, *Tg(mitfa:BRAF^V600E^)*, developed melanoma at a more rapid rate in a *p53^zdf1/zdf1^* mutant zebrafish background, showing genetic cooperation between the tumor suppressor p53 and B-Raf proto-oncogene (BRAF) pathways in melanoma development. Moreover, zebrafish with *p53* mutant background developed invasive and transplantable melanomas by 4 months of age. Importantly, zebrafish tumors were histopathologically similar to human melanoma [64]. Although *p53* is rarely mutated in human melanoma, the p53 pathway is often downregulated due to overexpression of its negative regulators MDM4 and MDM2 [50] or to inactivating mutations in *p14ARF* [65].

Although *BRAF* and *NRAS* mutations encompass 80% of the oncogenic drivers for human cutaneous melanoma, these are not sufficient to explain the aggressiveness of this tumor, suggesting cooperation with other somatic events during cancer development, including chromosomal aberrations. Computational analysis of human melanoma samples with the Genomic Identification of Signature Targets algorithm (GISTIC) [66], designed for analyzing chromosomal aberration in cancer, revealed amplification of chromosomal regions, with overexpression of interested genes at the RNA level [67]. To find genes that cooperate with oncogenic BRAF in melanoma initiation and progression, the chromosomal region 1q21 was scrutinized using the miniCoopR assay developed by Ceol, Houvras and Zon [68]. The miniCoopR assay is a transposon-based expression vector that encodes a *mifta* minigene under the *mitfa* promoter. 

In the work of Ceol et al., the transgenic fish developed by Patton et al. [64] *Tg(mitfa:BRAF^V600E^;p53^zdf1/zdf1^)* were bred with the *nacre* mutant, that carries an inactivating mutation of *mitfa*. In this way the authors generated fish lacking melanocytes and therefore unable to develop melanoma. Upon injection of the miniCoopR vector they observed a mosaic restoration of melanocytes and development of melanoma in the *Tg(mitfa:BRAF^V600E^)p53^zdf1/zdf1^/mitfa^−/−^.* The 30 genes of the 1q21 region were tested for their capacity of altering melanoma onset. The results of the screen showed that a single gene, SET domain bifurcated 1 (*SETDB1*), a histone H3K9 methyltransferase, is able to cooperate with the *BRAF^V600E^* mutation and promote melanoma development. Tumors co-expressing both *BRAF^V600E^* and *SETDB1* showed increased aggressiveness in *p53^zdf1/zdf1^* zebrafish. The 1q21 interval was later identified as a melanoma-susceptibility locus for familial melanoma, further demonstrating that SETDB1 is an oncogene in human melanoma [69]. The miniCoopR assay has also been used to validate the loss of 5-hydroxyl-methylcytosine (5-hmC) as an epigenetic hallmark of melanoma that correlates with clinical relapse-free survival. The melanoma epigenome showed lower levels of 5-hmC in comparison with benign nevi. Therefore, 5-hmC could be used as a biomarker with predictive and prognostic values. Loss of 5-hmC in melanoma is caused, at least partially, by the decreased expression of key enzymes that control 5-hmC deposition, such as IDH2 (isocitrate dehydrogenase 2) and TET (ten-eleven-translocation) family proteins [70].

#### 3.1.2. N-RAS^Q61K^

In 2009, Zon and colleagues generated two independent transgenic zebrafish lines with the melanocyte-specific promoter *mitfa,* which drives the expression of N-terminally *EGFP* tagged human *N-RAS^Q61K^* [71]. Expression of this oncogene led to hyperpigmentation throughout the body and disruption of the normal distribution of pigment cells. In a *p53^zdf1/zdf1^* mutant background, these animals presented aggressive melanoma, that were invasive and transplantable. Interestingly, gene set enrichment analysis (GSEA) was able to confirm correlation of up-regulated genes between human and zebrafish malignancies. Histopathology resembled the appearance and pathology of human melanoma. Melanoma developing in these *NRAS* transgenic lines is highly comparable with the human disease [71].

#### 3.1.3. H-RAS^G12V^

Hurlstone’s lab generated a transgenic line expressing mutant *HRAS* in melanocytes using the *mitfa* promoter [72]. This transgenic line was used in the chemical screen that is described below. Development of melanoma in these zebrafish is sporadic, but can be accelerated by activating mutations in the PI3K pathway, thus confirming the role of this pathway in malignant transformation downstream of HRAS [72].

Using the combinatorial GAL4-UAS system, a cross between a driver line containing the transactivator GAL4 under the *kita* promoter, and a responder line expressing human *HRAS^G12V^*, Mione’s lab developed a *p53*-independent melanoma model, that give rise to melanoma by 1–3 months of age [73]. Already at 3 dpf, transgenic larvae showed hyperpigmentation as a result of deregulated melanocyte growth. Between 2 and 4 weeks of age, condensed groups of melanocytes appeared throughout the tail and the double transgenic *et(kita:Gal4TA4,UAS:mCherry)^hzm1^; Tg(UAS:eGFP-HRAS_G12V)*^io006^, (name shortened in *kita-RAS^G12V^)* zebrafish, generating melanoma by one month of age that was also independent of PI3K signaling. The tumors had infiltrative behaviour, polyploid nuclei and expressed melanoma markers such as Tyrosinase, Melan-a, HMB45 and s100. In this way, they reproduced the main features of human melanoma from immunological, histological and molecular points of view, but over a shorter timeframe. The same experimental procedure was performed with the *mitfa* promoter driving *eGFP-HRAS^G12V^*. These animals developed melanoma starting from 4 months of age with a delay of 3 months and four times less efficiently than with the *kita* promoter, in spite of the same oncogene driver [73].

### 3.2. Chemical Screen to Identify Anti Melanoma Drugs

Chemical screens for melanoma-targeting drugs are performed with a variety of approaches (for detailed protocols see [74,75,76]). In order to identify suppressors of the neural crest progenitors that may give rise to melanoma, the Zon’s lab performed a large-scale chemical screen on zebrafish embryos [75]. To find initiating events in melanoma, they compared gene expression profiles of embryos and melanoma in *Tg(mitfa:BRAF^V600E^;p53^zdf1/zdf1^)* fish by GSEA. This comparison revealed an overlapping signature of 123 genes, containing markers of embryonic neural crest progenitors (*crestin*, *sox10*, *ednrb*) and melanocytes (*tyr*, *dct*). Several of these markers, including crestin, were then confirmed by in situ hybridization on adult zebrafish melanoma. These observations suggested that melanocytes return to a neural crest progenitor state when they become melanoma, and that BRAF^V600E^ could play a key role in this process [72]. By reasoning that chemicals effective in suppressing neural crest progenitors would also represent effective treatments for melanoma, they set up a large-scale screen using wild type zebrafish embryos and in situ hybridization to evaluate *crestin* expression. After screening 2000 compounds they found that one compound of unknown function, NSC210627, strongly reduced *crestin* expression [75]. 

NSC210627 was then found to have a similar structure to brequinar, a known dihydroorotate dehydrogenase (DHODH) inhibitor, and inhibited DHODH activity in vitro [77]. Leflunomide, another inhibitor of the metabolic enzyme DHODH and FDA-approved anti-arthritis drug, had an effect similar to NSC210627 in vivo, suppressing the expression of *crestin* and its targets, *mitfa* and *sox10*. Embryos treated with leflunomide lack melanocytes at 36–48 hpf, and exhibit a nearly complete loss of melanocyte progenitors at 24 hpf. Moreover, treatment of a human melanoma cell line with the active metabolite of leflunomide A771726, decreased proliferation in a dose-dependent manner. Co-treatment of melanoma cells with A771726 and a BRAF^V600E^ inhibitor had a synergistic effect in suppressing proliferation. A similar effect was observed in a mouse xenograft model, with the co-treatment resulting in nearly complete tumor regression in 40% of animals [75]. A phase I/II clinical trial with leflunomide in combination with the BRAF inhibitor vemurafenib (PLX4720) was initiated in patients with melanoma (Identifier: NCT01611675, clinicaltrial.gov). A new trial is now planned to combine leflunomide with dabrafenib and trematenib, BRAF, and MEK inhibitors [78].

In 2012, Patton and collaborators performed a chemical screen using changes in pigment cell phenotypes as readouts. To facilitate screening also in iridophores, experiments were performed with both wild type AB and *nacre* zebrafish [74]. The fish were screened at 48, 72 and 96 hpf for changes in melanocyte and iridophore quantity, location, and pigmentation-iridescence. At the time of treatment, neural crest development, which starts during the segmentation period at about 10 hpf, is still not initiated. They screened 1280 bioactive compounds from the Sigma LOPAC collection and the Enzo Life Sciences Screen-Well^TM^ Kinase and Phosphatase libraries. At the end, more than 50 compounds affecting pigmentation, migration and differentiation were identified [74]. 

In 2016, Hurlstone’s lab performed a chemical screen with 640 small molecules from a FDA approved library in combination with sub-optimal doses either of MEK inhibitor (PD184352) or PI3K-mTOR inhibitor (NVPBEZ235) using transgenic larvae overexpressing HRAS^G12V^ [79]. Ten hatched embryos at 2 dpf were incubated with the different drugs in a 24-well plate for 72 h. Embryos were then transferred to 96-well plates, melanin content was extracted and quantified through absorbance reading at 340 nm. Rapamycin was confirmed as a compound that could suppress melanoma growth in vivo and in vitro synergistically with MEK inhibitors (MEKi) and with PI3K/mTOR inhibitors. Two additional compounds, disulfiram and tanshinone were found to cooperate with MEKi to suppress the growth of transformed zebrafish melanocytes and showed activity toward cultured human melanoma cells [79].

### 3.3. Xenograft Models

One of the first attempts to establish zebrafish xenograft models involved the transplantation of fluorescently-tagged human metastatic melanoma cells into zebrafish blastula-stage embryos. The transplanted cells showed survival, proliferation, motility and were able to divide but did not develop tumors nor integrated into host organs. Cells were scattered in interstitial spaces throughout the embryo, indicating that melanoma cell lines are able to maintain their de-differentiated state and phenotype in zebrafish [80]. The methods for cancer cell transplantation in developing zebrafish are now standardized, and detailed protocols for different cancer cell lines can be found in [81,82].

In order to study interactions between cancer cells and embryonic progenitors, Hendrix and collaborators [83] transplanted fluorescently labeled human melanoma cells into zebrafish embryos at the blastula stage (3–5 hpf). Embryos were injected with C8161-GFP, an aggressive melanoma cell line, at the animal pole of the embryo or close to the blastoderm margin. Interestingly, depending on the site of engraftment, an abnormal outgrowth on the head (animal pole) or a duplication of the body axis (blastoderm margin) was observed. Whole-mount immune histochemistry (IHC) with antibody to beta-catenin and analysis at confocal microscopy were able to show that the ectopic masses on the head contained tissue comparable to axial mesoderm during the formation of the notochord. The same experiment was performed with the isogenic and less-aggressive, C8161-GFP melanoma cell line. In this case there was no growth of these ectopic structures. In the study, they also demonstrated that melanoma cells secrete Nodal, a highly conserved morphogen belonging to the transforming growth factor (TGF)-beta superfamily involved in embryonic axis formation. They showed that Nodal expression is present in human metastatic tumors but not in normal skin and that inhibition of Nodal is involved in the regression of melanoma cells toward a melanocytic phenotype, suggesting that Nodal could be a possible molecular target in melanoma progression [83]. 

In 2006, McGrath and co-workers optimized the protocol for transplanting WM-266-4 melanoma cells in zebrafish embryos. In order to assess proliferation and migratory pattern in zebrafish, human cells were labeled with CM-DiI, a fluorescent tracking dye [84]. WM-266-4 cell transplanted into the yolk sac at 2 dpf proliferated and formed masses, primarily in the regions surrounding the liver, pancreas and intestine. Masses were either embedded in tissue or protruding from the body. Moreover, after injection into the hindbrain ventricle at 2 dpf, human melanoma cells formed masses in the brain region. Whole-mount immunofluorescent staining followed by histology and 2-photon microscopy demonstrated the presence of activated zebrafish vascular endothelial cell that participated in the process of angiogenesis and formed new vessels in the masses formed by transplanted melanoma cells. Given the transparency of zebrafish embryos, the interaction between fluorescently tagged melanoma cells and zebrafish endothelial cells was visualized and tracked in real-time [84]. 

In 2008, White et al. crossed two already established mutant zebrafish lines, *nacre*, characterized by a complete loss of melanocytes and the *roy orbison* line, exhibiting a loss of the iridophores in order to create the *casper* mutant [85]. This mutant was used as recipient of xenografted tumor cells deriving from melanomas developing in *Tg(mitfa:NRAS^Q61K^:GFP);p53^zdf1/zdf1^* or *Tg(mitfa:BRAF^V600E^);p53^zdf1/zdf1^* zebrafish adult. Recipient fish were irradiated with an intensity of 25 Gray48 hours prior transplantation. Cells were transplanted into either the peritoneal cavity (IP) or via intracardiac injection (IC). By 5 days post-IP transplantation of NRAS:GFP cells, a large and highly pigmented intra-abdominal mass spreading also on the dorsal site appeared. After IC injection of NRAS:GFP cells, they proliferated and migrated along the needle tract but did not spread to more distal sites. Imaging of the same *Tg(mitfa:NRAS^Q61K^:GFP);p53^zdf1/zdf1^* fish over a 1 month period demonstrated gradual increase in tumor volume and development of metastasis in 37.5% of the recipient fish (*n* = 24) [85]. Thus, tumor growth, invasion, metastases and angiogenesis at an anatomic resolution not readily achievable in murine or other systems can be studied using the *casper* model.

Xenotransplantation of uveal melanoma cell lines was also performed in the zebrafish model by Snaar-Jagalska and collaborators [86]. Cell lines (92.1, Mel270, OMM1, OMM2.3, OMM2.5) derived from primary and metastatic UM, were injected in the yolk sac at 2 dpf. Cells were able to proliferate and migrate in the embryo. Cells from metastatic UM showed higher proliferation potential in comparison with those derived from the primary tumor. Moreover, to test whether this xenograft model was suitable for chemical screening three different anticancer drugs were used: quisinostat, MLN-4924 and dasatinib. Quisinostat is a pan-inhibitor of HDAC activity, MLN-4924 is an inhibitor of the neddylation pathway that leads to S-phase defects and apoptosis. Dasatinib is a BCR-ABL/Scr inhibitor. Treatments were performed on embryos transplanted with the OMM2.3 aggressive cell line or with the primary cell line 92.1. Embryos treated with dasatinib showed decreased growth only of primary UM transplanted cells, while the treatment of transplanted embryos with the broad-spectrum anticancer drugs MLN-4924 and quisinostat showed decreased growth and migration of both cell lines [86].

### 3.4. Perspectives on the Use of Zebrafish Melanoma Models for Tumor Heterogeneity and Cancer Immunotherapy

Nowadays, another major challenge in melanoma—leading inevitably to relapse in patients—is tumor heterogeneity. Zebrafish is emerging as a powerful model for the study of clonal dynamics. For example, in a recent paper by White and colleagues [87], which aimed to define genetic events that occur in the months after the expression of oncogenic BRAF, genomic DNA was isolated from ZMEL1—a cell line previously generated from a melanoma derived from a 6 months old *Tg*(*mitfa:BRAF^V600E^;p53^zdf1/zdf1^;mitfa^−/−^)* fish [68,88]—and compared to DNA derived from normal zebrafish muscle tissue. Whole genome sequencing demonstrated the appearance of 3000 new mutations. To test the presence of mutations occurring in drug resistant cells, ZMEL1 cells were cultured in the presence of the BRAF inhibitor vemurafenib for four months to give rise to a derivative resistant cell line ZMEL-R1. RNA from this line and from the parental ZMEL1 line was isolated and analyzed for differential gene expression as well as for mutation calling. Whole genome sequencing analysis revealed 3 new DNA mutations in *BUB1B*, *PINK1* and *COL16A1* genes and more than 800 genes differentially expressed between original ZMEL1 and ZMEL-R1 cells, especially those belonging to cyclic AMP (cAMP) and PKA signalling. Importantly, comparing both genomic landscape and gene expression profiles in human and zebrafish melanoma resistant to vemurafenib, the authors found conservation between zebrafish tumors and human melanoma [87]. These results highlight the importance of using zebrafish as a model for understanding mechanisms involved in genetic evolution in melanoma. In the future, specific patient mutations could be modeled in zebrafish and embryos of these lines used in chemical screens.

The function of the immune system in engineered zebrafish melanoma models has been poorly studied despite the fact that immunomodulatory drugs are already FDA approved for use in melanoma [89]. The most common immune modulatory agents used for treating melanoma tumor are represented by monoclonal antibody targeting CTLA-4-bearing T-cell (ipilimumab) or the PD-1 receptor (nivolumab) [89]. This delay in using zebrafish in studies of cancer immunity may be due to the lack of markers for the corresponding cells of the zebrafish immune system. Zebrafish CD4+ T cells, crucial for the effector immune response, were characterized by Hurlstone and collaborators in 2016 when they created a zebrafish transgenic model reporting the expression of CD4-1 [55]. Fish genomes contain multiple cd4-like paralogs called *cd4-1* and *cd4-2*. Using this model, they were able to demonstrate the presence of different population of CD4-1+ immune cells in fish melanoma: in the transgenic line *Tg(mitfa:eGFP,mitfa:RAS^V12^)^umc1^* they showed that CD4-1+ cells are able to infiltrate melanomas exactly as it happens in mammalian tumors [55]. These observations pave the way for the use of genetically modified zebrafish models of melanoma in the development of immune tools against cancer (Figure 1B).

## 4. Human Glioma and Relevant Target Pathways

Gliomas are the most common malignant primary tumors of the central nervous system (CNS) and affect about 2000 people in Europe each year with an overall age-adjusted incidence rate of 4.67–5.73 per 100,000 people [90]. Gliomas are classified by the World Health Organization (WHO) [91] according to histopathological and molecular features in grade I to IV, with grade I and II corresponding to slow-growing diffuse gliomas that are less aggressive, and grade III–IV corresponding to more aggressive and more malignant gliomas [91]. Grade IV gliomas are also known as glioblastoma multiforme (GBM), and are the most frequent and more malignant gliomas, characterized by a high degree of proliferation, tumor angiogenesis and chemoresistance [92].

A common feature of diffuse gliomas is extensive infiltrative growth into CNS parenchyma that prevents the full success of surgical removal, and requires the association with chemotherapy, usually performed with temozolomide (TMZ), as an adjuvant to radiotherapy [90,92]. TMZ is an imidazotetrazine alkylating agent, suitable for oral administration, used as standard first-line treatment for glioblastoma; although it provides a 2-year survival rate improvement from 15% to 26.6%, [93] at least 50% of TMZ treated patients do not respond to this drug. In addition, brain tumors are characterized by resistance to numerous drugs due to the poor penetrance of the blood-brain barrier (BBB) [94] and the failure to target glioma stem cells (GSCs) [95]. Combinatorial strategies involving different chemotherapeutic agent and pathway inhibitors are under investigation.

The high variability in the association between histological grade, cellular heterogeneity, progression and response to treatment (that entails the absence of prognostic indexes), have pushed glioma profiling through molecular characterization; indeed, GBM was the first cancer extensively sequenced by The Cancer Genome Atlas (TCGA) [96,97,98], bringing to a number of different classification of GBMs and other gliomas, arising from identification of markers [99,100,101,102].

Despite the fact that the mechanisms driving the progression of gliomas are still unknown, new therapeutic approaches are being developed to target multiple oncogenic signals that sustain brain cancer development.

Besides frequent-occurring mutations in IDH1 and IDH2 and TERT, there are four pathways that are physiologically and pathologically relevant to current glioma/glioblastoma therapies: (1) The P13K-PTEN-AKT-mTOR signaling pathway: In brain cells, this pathway is regulated by the epidermal growth factor (EGF), and its receptor, EGFR, both frequently overexpressed in GBM. P13K is altered in 79% of GBMs and it is often hyperactive as a result of loss of *PTEN*, a negative regulator of AKT [103]. (2) The *p53* signaling pathway: Inactivating mutations in *p53* can directly drive glioma formation through uncontrolled progression of cell cycle and by blocking apoptosis of cells with DNA damage [104,105,106]. (3) The Ras sarcoma (RAS)/MAPK signaling pathway: Although human RAS genes, including H-RAS, N-RAS and K-RAS are rarely mutated in glioma, the RAS/MAPK signaling cascade is a prime target for chemotherapy—this is due to numerous alterations of this pathway (for example: inactivation of NF-1, constitutive activation of BRAF and RAF1, MEK hyperactivation, abnormal activation of RAS by mutated growth factor receptors such as PDGFR or RTKs) that affect proliferative signaling and gliomagenesis [107,108]. (4) The cell cycle progression machinery: Critical regulators of cell growth are frequently mutated in human gliomas, including the tumor suppressor retinoblastoma (*pRB*), *CDK4* (activating mutations) and *CDKN2A* (inactivating mutations) with consequent overexpression of MDM2, an inhibitor of the tumor suppressor protein ARF (p14^ARF^). The homozygous deletion of locus *p16^INK4a^/p14^ARF^/p14^INK4b^* is one of the most frequent mutations in GBM [107,109,110].

These pathways are promising targets of new therapeutic approaches and are often chosen to establish transgenic models to investigate phenotypes and mechanisms leading to gliomagenesis (see below).

### 4.1. Zebrafish Glioma Models

In vivo tumor models provide essential information on how different genes, altered in a given glioma context, interact to drive tumor progression. Strategies to reproduce spontaneous glioma growth in zebrafish include genetically engineered models generated by germline/somatic modification of endogenous key glioma genes, transgenic and xenotransplantation models.

All zebrafish glioma models provide advantages for the monitoring of tumor infiltration, which is a feature of all brain tumors and help prognostication—this monitoring would require expensive and complex technologies in other systems.

A mutant/transgenic model to explore neurofibromin 1 (*NF1*) functions in brain tumorigenesis was generated in zebrafish [111]. The authors found two orthologues of *NF1* in zebrafish, *nf1a* and *nf1b* with high sequence similarities to human *NF1* and generated mutants of these genes by zinc finger nuclease (ZFN) and Target Induced Local Lesions in Genome (TILLING). *nf1a+/−/nf1b−/−* mutants in the *p53^zdf1/zdf1^* background developed brain tumors in 33 weeks, with characteristic markers (sox10/Olig2/Gfap) of diffuse high-grade gliomas and hyperactivation of ERK and mTOR pathways, similar to mouse models and human gliomas. The penetrance of these tumors in the triple mutants was compared with the penetrance of malignant peripheral nerve sheath tumors in *p53* mutants (28%) and showed to be higher (62%) [111]. In another report, overexpression of a dominant active form of AKT, DA-AKT1, under the *ptf1* promoter, initiates gliomagenesis in the cerebellum with high frequency (36.6% and 49% at 6 and 9 months, respectively). The induction of tumorigenesis was accelerated by co-expression of a dominant active (DA) form of RAC1, a protein involved in the migration and invasion of glioma cells (62% and 73.3% at 6 and 9 months, respectively). Moreover, DA-RAC1 also increased histological grade and invasiveness of brain tumors [112]. In this DA-AKT1 induced glioma model, the authors observed an upregulation of p21, cyclin D1, and survivin1-2 [112]. AKT-PI3K signaling pathway is activated in 90% of glioblastoma, and this model could be useful to study small molecule targeting the AKT pathway in a safe way.

The constitutive expression of human *AKT1* together with zebrafish Smoothened (*Smoa1*), an oncogene involved in Shh signaling, led to glioblastoma-like tumors in the brain, retina and spinal cord [113]. The same group reported that the expression of human *KRAS^G12V^* under two different neural promoter—*krt5* and *gfap*—induced high-grade brain tumors. The use of the two different promoters led to different tumor types: the *gfap* promoter drove expression in CNS with 50% incidence of tumor development; the *krt5* promoter induced aggressive nerve sheath tumors, supporting the idea that the origin of the tumor initiating cells affect tumor cell types [114]. Oncogenic expression of KRAS was studied also in inducible transgenic lines using TetOn system to test the effects of UO126, a strong MEK inhibitor, on ERK phosphorylation [114]. The results showed a suppression of mitotic-proliferative effects of oncogenic KRAS in zebrafish embryos. Recently, a new model to study brain tumor development was established by the Mione’s lab, through germline or somatic expression of *HRAS^G12V^* or other oncogenes (*YAP^S5A^*, *Xmrk*, *AKT*, *KRAS^G12V^*, *BRAF^V600E^*, *EGFRvIII*) under the control of the *zic4* enhancer [115]. Oncogenic RAS induced proliferation of neural progenitor cells, with massive brain tumors and reduced survival—the effects were highly reproducible in germline models, whereas somatic expression showed differences in tumor types associated with the area of origin of the somatic cells expressing the oncogene(s), providing a useful model for the study of the mechanisms that promote the progression from benign lesion to aggressive tumors [115].

Molecular studies of progressing brain tumors showed that the activation of YAP signaling kicked off at around 3 weeks, only in those tumors that progressed to malignant glioma. RAS/MAPK signaling instead induced both benign and aggressive brain tumors. Analysis of global RNA expression allowed to classify these zebrafish brain tumors as similar to the human mesenchymal GBM subtype, providing a defined model for preclinical screening and drug discovery for this very aggressive GBM subtype [115].

### 4.2. Human Glioma Transplantation in Zebrafish Embryos

A high impact technique developed in zebrafish is the orthotopic transplantation of human glioma cells in vivo. This technique offers the chance to visualize tumor development and tumor interactions with microenvironment at single-cell level in real time [116,117], an approach that in rat/mouse models requires sophisticated equipment and complex surgical techniques. A further advantage of zebrafish brain orthotopic xenotransplantation models is the late development of the BBB, which is completed only at 15 dpf [118], thus allowing several drug treatments before that stage.

Xenotransplantation of cancer cells in the brain can be performed at different stages, including in 30-day-old fish, but requires immunosuppression [119,120]. Nevertheless, implantation of human glioma cells in zebrafish embryos at 2 dpf showed a similar phenotype to mouse xenotransplantation [120]. In several studies, glioma cell lines as U251, U87 or CD133+ U87 stem-like cells (GSLC) were transplanted into the center of the yolk mass at 2 dpf [121,122,123] and analyzed for survival, proliferation and invasion. The movement of engrafted CD133^+^ U87 cells from the injection point showed that GBM cells with stem cell properties were more invasive than were the cells that lacked these properties.

Angiogenesis and the signaling pathway involved in glioma cell differentiation were studied in orthotopic xenotransplants of glioma cells into zebrafish brain [124,125]. Recently, Beattie and colleagues described a standardized method for intracranial transplantation of glioblastoma cells (from GBM9 neurospheres and X12 adherent stem and differentiated cells) in the proximity of the midbrain-hindbrain boundary of 36 hpf zebrafish embryos. In this work, the authors characterized the model extensively: the number of transplanted cells, survival test, proliferative capacity with ki67 immunostaining, presence of SOX2+ stem cells and GFAP-vimentin expression [126]. The approach is now used routinely for testing combinatorial drug treatments in GBM [127].

Another example of a well-established transplantation method of human U87 and U251 glioblastoma cells into the optic tectum was described in the transgenic *Tg(mpeg1:EGFP)^gl222^* line (macrophages/microglia marked with EGFP), to study the response of the entire microglial network during glioma colonization in real-time in the living brain [128].

To investigate the presence of tumor-initiating cells in stem cells cultures derived from high-grade pediatric brain tumors, cells were transplanted into zebrafish and mice [129]. Implanted cells were able to initiate tumors showing stemness properties. Moreover, experiments of co-transplantation of mesenchymal stem cells (MSCs) and U87/U373 showed a contribution of MSCs to GBM tumoral heterogeneity and tumor invasion [130,131].

### 4.3. Glioma Zebrafish Models and Therapeutic Insights

Although the mechanisms of gliomagenesis and progression remain largely unknown, recent advances in tumor modelling have directed the development of new therapeutic approaches.

Zebrafish xenograft approaches are frequently used to visualize several aspects of glioma in combination with transgenic models and drug treatments. Three studies showed how transplant of U251 or U87 [121,122,123] glioblastoma cells in a zebrafish transgenic model used to visualize blood vessels *Tg(fli1:EGFP)^y1^* [132], could be useful to evaluate the sensitivity to therapeutic treatments in relation to tumor angiogenesis and invasiveness.

In these models, cells proliferate forming tumors and recruiting blood vessels; the amount of neovessel formation allowed to evaluate the angiogenic potential of tumor cells as well as the effect of new treatments. Irradiation in the presence of TMZ decreased the survival of U251 cells growing within zebrafish embryos [122]; similarly, a small molecule identified after an in vitro chemical screen, NS123 (40-bromo-30-nitropropiophenone) enhanced the effect of ionizing radiation [121] on these cells. U87 GBM cells transplanted in the yolk sac were used to test anti-angiogenic effects of Axitinib, Suntinib and Vatalani, drugs which inhibited tumor-induced vessel formation, and the therapeutic potential of the synthetic dl-nordihydroguaiaretic acid compound, Nordy, as an anti-GSC agent [133]. More recently, the *Tg(fli1:EGFP)^y1^* line was used to test the efficacy of anti-vascular endothelial growth factor (VEGF) siRNAs, a promising therapeutic alternative for the treatment of brain tumors. In particular, the study evaluated the ability of exosome-entrapped siRNA to cross the BBB and inhibit VEGF expression in U-87 glioblastoma cells xenotransplanted in zebrafish embryos at 3 dpf [56].

The previously described GBM9 neurospheres transplantation was later used to define how glioblastoma populations respond to the TMZ chemotherapeutic agent, and the role of putative cancer stem cells in drug resistance and in tumor regrowth after treatment [127].

Gilberston and colleagues [119] tested two chemotherapeutic drugs—the cytotoxic antimetabolite 5-fluorouracil (5-FU) and tyrosine kinase inhibitor Erlotinib—in mouse brain tumor cells orthotopically implanted in 30-day-old zebrafish immunosuppressed with dexamethasone. In this contest, rejection was not observed and brain tumors developed, showing histological features of parental tumors, and the ability to form metastasis. Moreover, chemotherapy with 5-FU and Erlotinib was able to inhibit tumor proliferation. Treatment with Erlotinib blocked ERBB2 signaling as previously showed in mouse [134], and allowed to reduce the dosage of 5-FU, with a 50% reduction in tumor size after only two days of treatment. These experiments prove how these xenotransplantation models could be powerful tools in testing drug combination efficacy [119].

A high-throughput screening (HTS) of compound targeting glioma growth in zebrafish, has not yet been performed and could provide important information on drug efficacy and their ability to cross the BBB, with immediate benefit for the development of new therapeutics in GBM.

A complementary approach to identify compounds that prevent pediatric brain tumor growth in vivo was described by Stewart and colleagues [135]. In this study, embryonic brain tumors were generated by orthotopic transplantation of cells derived from genetically engineered zebrafish models of embryonal brain tumors into hundreds of recipient embryos at 2 dpf. The original donors were generated through the expression of oncogenic or wild type human NRAS driven by the *sox10* promoter in a mixed *p53^zdf1/zdf1^* and *mitfa^w2^* [136] background. Histological and molecular analysis of the primary tumors developed in the CNS after 6 weeks, showed conservation of features with human embryonic brain tumors. Elevated expression of *sox10/olig2* and activation of the RAS/MAPK pathway were also reported. Then, single-cells suspensions were harvested and injected into the fourth ventricle of 2 dpf embryo allowing direct visualization of forming tumors, thanks to the fusion of NRAS with mCherry, tumors spread to the brain by 17 days post transplantation. Transplanted embryos were subjected to a rapid drug-screening to test if RAS/MAPK pathway inhibitors could prevent tumor spreading. Three dpf embryos were treated with RAS/MAPK inhibitors for 5 days and the tumor area was quantified. Treatment with AZD6244 and U0126, two MEK inhibitors, induced significant shrinkage of tumor spreading after 5 days of treatment. To validate these promising results, the authors treated juvenile fish with primary tumors with AZD6244 for 8 weeks, showing higher post treatment survival, with only 21% of fish having tumors in contrast to 88% of fish treated with dimethyl sulfoxide (DMSO). These results showed that MEK inhibitors were remarkably powerful to decrease tumor growth [135].

### 4.4. Translational Impact of Zebrafish Models in Glioma Research

Glioma represent 80% of malignant brain tumors and their successful treatment depends on location, exact molecular type and age of the patient [90,91,92,137]. Clinical investigation and novel concepts need to be tested to identify effective targets. To this aim, translational studies can facilitate the discovery of combinatorial strategies involving different chemotherapeutic agents targeting different pathways.

The previously described zebrafish models showed how they could be used in molecular studies and in preclinical screenings of drugs targeting aggressive GBM subtypes. At the same time, it is possible to directly visualize changes in the microenvironment arising from tumor-induced angiogenesis and immune responses, using combinations of fluorescent transgenic lines marking immune cells and blood vessels [121,122,123,128].

The development of increasingly reproducible xenotransplantation protocols of human and mouse glioblastoma cells has provided new information about the role of putative glioma cancer stem cells in tumor relapse and drug resistance.

In this respect, a primary brain cancer model developed after xenotransplantation was recently used to study how to safely deliver siRNA across the BBB [56], with the goal of knocking down a target gene with high specificity and low toxicity (Figure 1C). Thus, the zebrafish stands up as the model of choice for the development of nanotechnological tools for genetic engineering to be used in vivo.

## 5. Studying Endocrine Tumors Using Zebrafish

The endocrine system is composed of a variety of glands such as the pituitary, parathyroid, thyroid, adrenal, endocrine pancreas, and the diffuse neuroendocrine system. Neuroendocrine cells are present in the gastrointestinal tract, lung, thymus, and ovary. The zebrafish endocrine system is similar to its human counterpart in both the structure of the organs and the transcription factors involved in the organ specifications and formation [138]. To study the molecular mechanisms at the origin of endocrine and neuroendocrine tumors, zebrafish models have been generated.

### 5.1. Pituitary Tumor

The first model of pituitary tumors was generated in 2011 by Melmed and coworker [139], with the aim of studying Cushing disease. This syndrome is characterized by increased secretion of adrenocorticotropic hormone (ACTH) from adenomas of the anterior pituitary gland. Transgenic fish were generated by expressing zebrafish *pttg* (Pituitary tumor-transforming 1) in the pituitary proopiomelanocortin (POMC) lineage (corticotrophs and melanotrophs). This transgenic model developed an early embryonic phenotype reflective of corticotroph tumors featuring corticotrophs expansion with partial glucocorticoid resistance. Thanks to this early phenotype, the authors performed a small chemical screen testing CDK inhibitors and found that Roscovitine, a CDK2/cyclin E inhibitor, reversed corticotrophs expansion in embryos. Importantly, these results were also confirmed in mice, in which orally administered Roscovitine decreased ACTH and corticosterone levels, while blocking tumor growth [139]. Based on the observations that Roscovitine suppresses pituitary corticotroph tumor and ACTH production in patients with Cushing disease, a phase II clinical trial is currently recruiting patients with Cushing disease (Identifier: NCT02160730), thus supporting the use of zebrafish in preclinical drug discovery also for these tumors.

### 5.2. Papillary Thyroid Carcinoma

The first transgenic model of zebrafish PTC has been recently generated in Houvras’ lab [57] by co-expressing the human oncogene BRAF^V600E^ and the fluorescent protein Td-Tomato in thyrocytes through a thyroid specific promoter, thyroglobulin. This model presented an embryonic phenotype characterized by developmental defects of thyroid structure and absence of hormone T4 production, allowing the study of initial events of transformation. Fluorescent thyrocytes were sorted from embryos to analyze gene expression induced by oncogenic BRAF. Two signatures, TGF-beta and epidermal to mesenchymal transition (EMT), were found differentially expressed in transformed thyrocytes compared to control. In particular, the expression of one transcription factor involved in EMT, *twist3*, was found upregulated in BRAF^V600E^-expressing thyrocytes. Loss of *twist3* expression using Crispr/Cas9 technology restored normal thyroid structure and T4 production, demonstrating the key role of this transcription factor in thyroid cancer initiation. Moreover, treatment of BRAF-expressing embryos with a combination of BRAF and MEK inhibitors was able to restore follicular structure, demonstrating the suitability of this model to be used in chemical screens to investigate BRAF-mediated pathways. Another important observation that came out from this study was that thyroid tumors in adult zebrafish harbor a gene expression signature that stratifies disease recurrence in patients with PTC, identifying patients with significant lower risk of relapse. Besides having clinical significance, these results demonstrated a high similarity between human and zebrafish thyroid cancer [57].

In parallel with the generation of a zebrafish model of PTC, xenotransplantation of human derived PTC cells has been performed. To investigate the proangiogenic potential of PTC stem cells, spheroids (that are enriched of stem cell) obtained from patients with PTC were transplanted into *Tg(fli1a:EGFP)^y1^* transgenic fish, that have green fluorescent vasculature [132]. Spheroids were able to activate neoangiogenesis processes. This in vivo model could be a useful platform for testing anticancer drugs for their ability to inhibit tumor growth and angiogenesis [140].

### 5.3. Neuroendocrine Tumors

To study the biology of neuroendocrine tumors in vivo, a model based on patient-derived xenografts of neuroendocrine tumor cells in zebrafish *Tg(fli1a:EGFP)^y1^* fish was established [141]. Both pituitary adenoma and neuroendocrine tumor primary cell cultures were obtained from surgical samples. Patient-derived xenograft (PDXs) of neuroendocrine tumors showed both pro-angiogenic and invasive behaviors within 48 h post infection (hpi), and these effects were evident as early as 24 hpi. This model is a promising method for the neuroendocrine tumors preclinical research, because the procedure of injecting cancer cells in zebrafish embryos is simple and small tumor implants (100 cells/embryos) are sufficient to study tumor growth. This is a big advantage because tumor cell availability is often limited, due to the small size of this type of tumor. Once again, these patient-derived cancer cells can be injected into *Tg(fli1a:EGFP)^y1^* transgenic fish to evaluate the anti-angiogenic effects of drugs [141].

### 5.4. Summary and Translational Impact of Zebrafish Models in Endocrine Tumors Research

In summary, zebrafish have been revealed as a powerful animal model for the study of the biology of endocrine tumors, thanks to the generation of transgenic lines developing endocrine tumors and to xenotransplantation of human cancer cells into embryos. Gene expression analysis of zebrafish PTCs showed that these tumors harbor a gene expression signature that stratify disease recurrence in patients with PTC, identifying a class of patients with lower risk of relapse (Figure 1D). Moreover, a chemical screen performed on transgenic fish developing pituitary tumors revealed the efficacy of Roscovitine to reversed corticotroph cell expansion in embryos. A clinical translation of this research is demonstrated by the fact that a phase II clinical trial is currently recruiting patients with Cushing syndrome to test the effects of Roscovitine [139].

## 6. Conclusions and Future Perspectives

In this review, we discussed the power of zebrafish in finding new targeted and personalized treatments for leukemia, melanoma, glioma and endocrine tumors, which should lead to better clinical outcomes and less toxicity for patients. A few examples that highlight novel promising aspects of the use of zebrafish cancer models in translational research are shown in Figure 1, focusing on leukemia (Figure 1A), melanoma (Figure 1B), brain cancer (Figure 1C) and thyroid cancer (Figure 1D). Moreover, a summary of chemical screens and transplantation experiments performed in zebrafish with their major findings are listed in Table 1 and Table 2. However, besides many advantages, there are still some challenges in the use of this model organism in cancer biology. For example, the difference in temperatures favorable to human cells and zebrafish maintenance is still an obstacle for long term studies of PDXs. In fact, once implanted into zebrafish embryos, human cells stop growing when kept at 28 °C, which is the temperature at which embryos and adult zebrafish are kept. Indeed, with the exception of one study [126], none of the studies show the ability of human cancer cells to proliferate in zebrafish embryos after implantation. Future studies should address this problem, for example by generating zebrafish mutants or transgenic lines able to adapt to higher temperatures. These lines could be generated by the manipulation of genes involved in metabolism using Crispr/Cas9 technology. The availability of these lines and the use of the immune-compromised lines generated by Langenau’s lab [37] will lead to the use of zebrafish in more PDX studies.

## Figures and Tables

**Table 1:** Chemical screens performed in zebrafish and their major findings.

Tumor Type	Fish Line	Compounds Screened	Read out	Hits	Major Findings	Clinical Trials	References
Leukemia	wild type *AB* strain	2480	in situ hybridization for runx1 and cmyb	PGE2	PGE2 is a regulator of HSCs number	Phase I	[38]
	*Tg(lck:lck-EGFP)*	26400	T-cells (GFP+)	Lenaldekar (LDK)	LDK delays mitosis and inhibits PI3K/AKT/mTOR pathway		[41]
	*Tg(rag2:EGFP-mMyc)*	4880	T-cells (GFP+)	Perphenazine (PPZ)	PPZ activates the tumor suppressor protein phosphatase 2A (PP2A)		[43]
Melanoma	*AB* and *nacre mutant*	1280	changes in pigment cell phenotype	More than 50	More than 50 compound affect pigmentation, migration and differentiation		[74]
	*Tg(mitfa:BRAF^V600E^); p53^zdf1/zdf1^*	2000	in situ hybridization for crestin	NSC210627 and Leflunomide	Crestin is aberrantly expressed in tumor cells, leflunomide and NSC210627 suppress crestin expression	Phase I/II	[75]
	*Tg(mitfa:HRAS^V12^; mitfa:GFP)*	640	melanin quantification through absorbance reading at 340 nm	Rapamycin, disulfiram, tanshinone	Rapamycin, disulfiram and tanshinone cooperate with MEKi to suppress growth of melanocytes		[79]
Endocrine tumors	*Tg:Pomc-Pttg;Pomc-eGFP*	CDK inhibitors	pituitary cells (GFP+)	Roscovitine	Roscovitine reverse corticotrophs expansion in embryos	Phase II	[139]

PEG2; prostaglandin E2; HSC; hematopoietic stem cells; PI3K/AKT/mTOR: phosphoinositide 3-kinase/AKT/ mechanistic target of rapamycin; CDK: cyclin-dependent kinase.

**Table 2:** Transplantation experiments performed in zebrafish and their major findings.

Tumor Type	Transplanted Cells	Recipient Fish	Drug Treatments	Major Findings	References
Leukemia	zf T-ALL (GFP+)	wild type *AB* strain irradiated	-	Leukemic cells infiltrate region adjacent to the olfactory bulb	[24]
	zf T-ALL (GFP+)	CG1 strain	-	T-ALLs can be initiated from a single cell; T-ALLs exhibit wide differences in tumor-iniating potential	[32]
	zf T-ALL (GFP+)	CG1 strain	-	Notch signaling expands a population of pre-malignant thymocytes in T-ALL	[33]
	zf T-ALL (several fluorescent clonal markers)	CG1 strain	MK2206+ dexamethasone	AKT pathway increases the number of LPC; MK2206+dexamethasone kill T-ALL cells	[34]
	zf T-ALL (several fluorescent clonal markers)	*prkdc mutant*	-	Clonal dominance emerges as a consequence of neutral stochastic drifts	[37]
	hu leukemic cell lines (K562, K562-R, Jurkat, NB4)	wild type *AB* strain	anti-leukemic drugs	Imatinib and Oxaphorines decrease the leukemic burden in xenografted animals	[44]
Melanoma	hu metastatic melanoma cells (GFP+)	wild type *AB* strain	-	Metastatic melanoma cell lines are able to retain their dedifferentiated state	[80]
	hu metastatic melanoma cells (C8161) (GFP+)	wild type *AB* strain	-	Nodal expression is involved in the regulation of melanoma progression	[83]
	hu metastatic melanoma cells (WM-266-4) (CM-DiI+)	wild type *AB* strain	-	Transplanted cells proliferate, migrate and stimulate angiogenesis	[84]
	zf melanoma cells	*casper* irradiated fish	-	Tranplanted cells can be tracked in adult transparent *casper* fish	[85]
	hu primary and metastatic uveal melanoma cell lines	*Tg(fli1a:EGFP)*	Quinostat, MLN-4924, dasatinib	Quinostat and MLN-4924 decrease migration and proliferation of primary and metastic UM cells	[86]
Glioma	mo primary brain tumor cells (RFP+)	*Tg(fli1a:EGFP)*	5-FU, Erlotinib	5-FU and Erlotinib combination is able to inhibit tumor proliferation	[119]
	hu U251 (RFP+)	*Tg(fli1a:EGFP)*	NS-123	NS-123 synergies the effects of ionizing radiation on inhibition of tumor growth	[121]
	hu U251 (RFP+)	*Tg(fli1a:EGFP)*	Temozolomide	Experiments done to test the efficacy of combined temozolomide and radiation	[122]
	hu CD133+ U87 (RFP+)	*Tg(fli1a:EGFP)*	AG-L-66085	U87 GSCs have enhanced expression MMP-9, which regulates glioma invasion	[123]
	hu U87	*Tg(fli1a:EGFP)*	-	Calpain 2 expression is required for glioblastoma cell invasion	[124]
	hu primary GBM cells	*Tg(7xTCF-Xla.Siam:GFP)*	-	WNT pathway activation promotes neuronal differentiation	[125]
	hu GBM9, hu X12 (GFP+)	wild type *ABLF*	Temozolomide, bortezomib	Standardization of orthotopic xenograft methods; chemotherapy changes tumor cell heteregeneity	[126,127]
	hu U251, U87 (mCherry+)	*Tg(mpeg1:EGFP);irf8^-/-^*	BLZ945	Microglia promotes human glioblastoma cell growth	[128]
	hu primary pediatric brain tumor cells (RFP+)	wild type *AB* strain	-	Stem cells derived from pediatric brain tumor are tumorigenic in zebrafish	[129]
	hu MSC (DiO), hu U373 (eEGFP), hu U87 (dsRed+)	*wild type AB strain*	-	MSCs contribuite to GBM tumoral heterogeneity and invasion	[130]
	hu GSCs U87 (RFP+)	*Tg(fli1a:EGFP)*	Nordy, Axitinib, Suntinib, Vatalani	Nordy suppresses the proliferation of GSCs	[133]
	hu U87MG (Cell Brite® DiD)	*Tg(fli1a:EGFP)*	-	anti-VEGF siRNAs cross the BBB and inhibit VEGF expression in a xenotransplanted brain tumor	[56]
	zf primary brain tumor cells	*nacre*, *p53*^zdf1/zdf1^	AZD6244, U0126	MEK inhibitors decrease brain tumor growth	[135]
Endocrine tumors	hu PTC spheroids	*Tg(fli1a:EGFP)*	-	Spheroids are able to activate neoangiogenesis	[140]
	hu neuroendocrine tumors	*Tg(fli1a:EGFP)*	-	Neuroendocrine tumors are pro-angiogenic and invasive	[141]

T-ALL: T-cell acute lymphoblastic leukemia; UM: uveal melanoma; GBM: glioblastoma multiforme; RFP: red fluorescent protein; GSC: glioma stem cells; WNT: Wingless-related integration site; MSC: mesenchymal stem cells; PTC; papillary thyroid carcinoma.

## References

[1] Lieschke G.J., Currie P.D. (2007). Animal models of human disease: Zebrafish swim into view. Nat. Rev. Genet..

[2] Howe K., Clark M.D., Torroja C.F., Torrance J., Berthelot C., Muffato M., Collins J.E., Humphray S., McLaren K., Matthews L. (2013). The zebrafish reference genome sequence and its relationship to the human genome. Nature.

[3] Zhao S., Huang J., Ye J. (2015). A fresh look at zebrafish from the perspective of cancer research. J. Exp. Clin. Cancer Res..

[4] Veinotte C.J., Dellaire G., Berman J.N. (2014). Hooking the big one: The potential of zebrafish xenotransplantation to reform cancer drug screening in the genomic era. Dis. Model. Mech..

[5] Wiley D.S., Redfield S.E., Zon L.I. (2017). Chemical screening in zebrafish for novel biological and therapeutic discovery. Methods Cell Biol..

[6] Zhu S., Thomas Look A. (2016). Neuroblastoma and Its Zebrafish Model. Adv. Exp. Med. Biol..

[7] Hwang K.L., Goessling W. (2016). Baiting for Cancer: Using the Zebrafish as a Model in Liver and Pancreatic Cancer. Adv. Exp. Med. Biol..

[8] Jing L., Zon L.I. (2011). Zebrafish as a model for normal and malignant hematopoiesis. Dis. Model. Mech..

[9] Kwan W., North T.E. (2017). Netting Novel Regulators of Hematopoiesis and Hematologic Malignancies in Zebrafish. Curr. Top. Dev. Biol..

[10] Paik E.J., Zon L.I. (2010). Hematopoietic development in the zebrafish. Int. J. Dev. Biol..

[11] Stachura D.L., Traver D. (2011). Cellular dissection of zebrafish hematopoiesis. Methods Cell Biol..

[12] American Cancer Society (2017). Cancer Facts & Figures 2017.

[13] Terwilliger T., Abdul-Hay M. (2017). Acute lymphoblastic leukemia: A comprehensive review and 2017 update. Blood Cancer J..

[14] Oliveira M.L., Akkapeddi P., Alcobia I., Almeida A.R., Cardoso B.A., Fragoso R., Serafim T.L., Barata J.T. (2017). From the outside, from within: Biological and therapeutic relevance of signal transduction in T-cell acute lymphoblastic leukemia. Cell. Signal..

[15] Mullighan C.G. (2012). The molecular genetic makeup of acute lymphoblastic leukemia. Hematol. Am. Soc. Hematol. Educ. Progr..

[16] Bhatnagar B., Garzon R. (2014). The use of molecular genetics to refine prognosis in acute myeloid leukemia. Curr. Hematol. Malig. Rep..

[17] Roberts K.G., Mullighan C.G. (2015). Genomics in acute lymphoblastic leukaemia: Insights and treatment implications. Nat. Rev. Clin. Oncol..

[18] Liu J., Zhou Y., Qi X., Chen J., Chen W., Qiu G., Wu Z., Wu N. (2017). CRISPR/Cas9 in zebrafish: An efficient combination for human genetic diseases modeling. Hum. Genet..

[19] De Kouchkovsky I., Abdul-Hay M. (2016). Acute myeloid leukemia: A comprehensive review and 2016 update. Blood Cancer J..

[20] Hunger S.P., Mullighan C.G. (2015). Acute Lymphoblastic Leukemia in Children. N. Engl. J. Med..

[21] Litzow M.R., Ferrando A.A. (2015). How I treat T-cell acute lymphoblastic leukemia in adults. Blood.

[22] Gottlieb A.J., Weinberg V., Ellison R.R., Henderson E.S., Terebelo H., Rafla S., Cuttner J., Silver R.T., Carey R.W., Levy R.N. (1984). Efficacy of daunorubicin in the therapy of adult acute lymphocytic leukemia: A prospective randomized trial by cancer and leukemia group B. Blood.

[23] Scavino H.F., George J.N., Sears D.A. (1976). Remission induction in adult acute lymphocytic leukemia. Use of vincristine and prednisone alone. Cancer.

[24] Langenau D.M., Traver D., Ferrando A.A., Kutok J.L., Aster J.C., Kanki J.P., Lin S., Prochownik E., Trede N.S., Zon L.I. (2003). Myc-induced T cell leukemia in transgenic zebrafish. Science.

[25] Yeh J.-R.J., Munson K.M., Elagib K.E., Goldfarb A.N., Sweetser D.A., Peterson R.T. (2009). Discovering chemical modifiers of oncogene-regulated hematopoietic differentiation. Nat. Chem. Biol..

[26] Langenau D.M., Feng H., Berghmans S., Kanki J.P., Kutok J.L., Look A.T. (2005). Cre/lox-regulated transgenic zebrafish model with conditional myc-induced T cell acute lymphoblastic leukemia. Proc. Natl. Acad. Sci. USA.

[27] Feng H., Langenau D.M., Madge J.A., Quinkertz A., Gutierrez A., Neuberg D.S., Kanki J.P., Look A.T. (2007). Heat-shock induction of T-cell lymphoma/leukaemia in conditional Cre/lox-regulated transgenic zebrafish. Br. J. Haematol..

[28] Gutierrez A., Grebliunaite R., Feng H., Kozakewich E., Zhu S., Guo F., Payne E., Mansour M., Dahlberg S.E., Neuberg D.S. (2011). Pten mediates Myc oncogene dependence in a conditional zebrafish model of T cell acute lymphoblastic leukemia. J. Exp. Med..

[29] Ellisen L.W., Bird J., West D.C., Soreng A.L., Reynolds T.C., Smith S.D., Sklar J. (1991). TAN-1, the human homolog of the Drosophila notch gene, is broken by chromosomal translocations in T lymphoblastic neoplasms. Cell.

[30] Suresh S., Irvine A.E. (2015). The NOTCH signaling pathway in normal and malignant blood cell production. J. Cell Commun. Signal..

[31] Chen J., Jette C., Kanki J.P., Aster J.C., Look A.T., Griffin J.D. (2007). NOTCH1-induced T-cell leukemia in transgenic zebrafish. Leukemia.

[32] Smith A.C.H., Raimondi A.R., Salthouse C.D., Ignatius M.S., Blackburn J.S., Mizgirev I.V., Storer N.Y., de Jong J.L.O., Chen A.T., Zhou Y. (2010). High-throughput cell transplantation establishes that tumor-initiating cells are abundant in zebrafish T-cell acute lymphoblastic leukemia. Blood.

[33] Blackburn J.S., Liu S., Raiser D.M., Martinez S.A., Feng H., Meeker N.D., Gentry J., Neuberg D., Look A.T., Ramaswamy S., Bernards A. (2012). Notch signaling expands a pre-malignant pool of T-cell acute lymphoblastic leukemia clones without affecting leukemia-propagating cell frequency. Leukemia.

[34] Blackburn J.S., Liu S., Wilder J.L., Dobrinski K.P., Lobbardi R., Moore F.E., Martinez S.A., Chen E.Y., Lee C., Langenau D.M. (2014). Clonal evolution enhances leukemia-propagating cell frequency in T cell acute lymphoblastic leukemia through Akt/mTORC1 pathway activation. Cancer Cell.

[35] Tang Q., Abdelfattah N.S., Blackburn J.S., Moore J.C., Martinez S.A., Moore F.E., Lobbardi R., Tenente I.M., Ignatius M.S. (2014). Optimized cell transplantation using adult rag2 mutant zebrafish. Nat. Methods.

[36] Elnour I.B., Ahmed S., Halim K., Nirmala V. (2007). Omenn’s Syndrome: A rare primary immunodeficiency disorder. Sultan Qaboos Univ. Med. J..

[37] Moore J.C., Tang Q., Yordán N.T., Moore F.E., Garcia E.G., Lobbardi R., Ramakrishnan A., Marvin D.L., Anselmo A., Sadreyev R.I. (2016). Single-cell imaging of normal and malignant cell engraftment into optically clear prkdc-null SCID zebrafish. J. Exp. Med..

[38] North T.E., Goessling W., Walkley C.R., Lengerke C., Kopani K.R., Lord A.M., Weber G.J., Bowman T.V., Jang I.-H., Grosser T. (2007). Prostaglandin E2 regulates vertebrate haematopoietic stem cell homeostasis. Nature.

[39] Goessling W., Allen R.S., Guan X., Jin P., Uchida N., Dovey M., Harris J.M., Metzger M.E., Bonifacino A.C., Stroncek D. (2011). Prostaglandin E2 enhances human cord blood stem cell xenotransplants and shows long-term safety in preclinical nonhuman primate transplant models. Cell Stem Cell.

[40] Cutler C., Multani P., Robbins D., Kim H.T., Le T., Hoggatt J., Pelus L.M., Desponts C., Chen Y.-B., Rezner B. (2013). Prostaglandin-modulated umbilical cord blood hematopoietic stem cell transplantation. Blood.

[41] Ridges S., Heaton W.L., Joshi D., Choi H., Eiring A., Batchelor L., Choudhry P., Manos E.J., Sofla H., Sanati A. (2012). Zebrafish screen identifies novel compound with selective toxicity against leukemia. Blood.

[42] Langenau D.M., Ferrando A.A., Traver D., Kutok J.L., Hezel J.-P.D., Kanki J.P., Zon L.I., Look A.T., Trede N.S. (2004). In vivo tracking of T cell development, ablation, and engraftment in transgenic zebrafish. Proc. Natl. Acad. Sci. USA.

[43] Gutierrez A., Pan L., Groen R.W.J., Baleydier F., Kentsis A., Marineau J., Grebliunaite R., Kozakewich E., Reed C., Pflumio F. (2014). Phenothiazines induce PP2A-mediated apoptosis in T cell acute lymphoblastic leukemia. J. Clin. Investig..

[44] Pruvot B., Jacquel A., Droin N., Auberger P., Bouscary D., Tamburini J., Muller M., Fontenay M., Chluba J., Solary E. (2011). Leukemic cell xenograft in zebrafish embryo for investigating drug efficacy. Haematologica.

[45] Downing J.R. (1999). The AML1-ETO chimaeric transcription factor in acute myeloid leukaemia: Biology and clinical significance. Br. J. Haematol..

[46] Troke P.J.F., Kindle K.B., Collins H.M., Heery D.M. (2006). MOZ fusion proteins in acute myeloid leukaemia. Biochem. Soc. Symp..

[47] Borrow J., Shearman A.M., Stanton V.P., Becher R., Collins T., Williams A.J., Dubé I., Katz F., Kwong Y.L., Morris C. (1996). The t(7;11)(p15;p15) translocation in acute myeloid leukaemia fuses the genes for nucleoporin NUP98 and class I homeoprotein HOXA9. Nat. Genet..

[48] Ley T.J., Miller C., Ding L., Raphael B.J., Mungall A.J., Robertson A.G., Hoadley K., Triche T.J., Laird P.W., Cancer Genome Atlas Research Network (2013). Genomic and epigenomic landscapes of adult de novo acute myeloid leukemia. N. Engl. J. Med..

[49] Dombret H. (2011). Gene mutation and AML pathogenesis. Blood.

[50] Zhuravleva J., Paggetti J., Martin L., Hammann A., Solary E., Bastie J.-N., Delva L. (2008). MOZ/TIF2-induced acute myeloid leukaemia in transgenic fish. Br. J. Haematol..

[51] Yeh J.-R.J., Munson K.M., Chao Y.L., Peterson Q.P., Macrae C.A., Peterson R.T. (2008). AML1-ETO reprograms hematopoietic cell fate by downregulating *scl* expression. Development.

[52] Forrester A.M., Grabher C., McBride E.R., Boyd E.R., Vigerstad M.H., Edgar A., Kai F.-B., Da’as S.I., Payne E., Look A.T. (2011). NUP98-HOXA9-transgenic zebrafish develop a myeloproliferative neoplasm and provide new insight into mechanisms of myeloid leukaemogenesis. Br. J. Haematol..

[53] Alghisi E., Distel M., Malagola M., Anelli V., Santoriello C., Herwig L., Krudewig A., Henkel C.V., Russo D., Mione M.C. (2013). Targeting oncogene expression to endothelial cells induces proliferation of the myelo-erythroid lineage by repressing the Notch pathway. Leukemia.

[54] Zhang Y., Wang J., Wheat J., Chen X., Jin S., Sadrzadeh H., Fathi A.T., Peterson R.T., Kung A.L., Sweetser D.A. (2013). *AML1-ETO* mediates hematopoietic self-renewal and leukemogenesis through a COX/β-catenin signaling pathway. Blood.

[55] Dee C.T., Nagaraju R.T., Athanasiadis E.I., Gray C., Fernandez Del Ama L., Johnston S.A., Secombes C.J., Cvejic A., Hurlstone A.F.L. (2016). CD4-Transgenic Zebrafish Reveal Tissue-Resident Th2- and Regulatory T Cell-like Populations and Diverse Mononuclear Phagocytes. J. Immunol..

[56] Yang T., Fogarty B., LaForge B., Aziz S., Pham T., Lai L., Bai S. (2017). Delivery of small interfering RNA to inhibit vascular endothelial growth factor in zebrafish using natural brain endothelia cell-secreted exosome nanovesicles for the treatment of brain cancer. AAPS J..

[57] Anelli V., Villefranc J.A., Chhangawala S., Martinez-McFaline R., Riva E., Nguyen A., Verma A., Bareja R., Chen Z., Scognamiglio T. (2017). Oncogenic BRAF disrupts thyroid morphogenesis and function via twist expression. Elife.

[58] Schartl M., Larue L., Goda M., Bosenberg M.W., Hashimoto H., Kelsh R.N. (2016). What is a vertebrate pigment cell?. Pigment Cell Melanoma Res..

[59] Schadendorf D., Hauschild A. (2014). Melanoma in 2013: Melanoma—The run of success continues. Nat. Rev. Clin. Oncol..

[60] Schadendorf D., Fisher D.E., Garbe C., Gershenwald J.E., Grob J.-J., Halpern A., Herlyn M., Marchetti M.A., McArthur G., Ribas A. (2015). Melanoma. Nat. Rev. Dis. Primers.

[61] Hodis E., Watson I.R., Kryukov G.V., Arold S.T., Imielinski M., Theurillat J.-P., Nickerson E., Auclair D., Li L., Place C. (2012). A landscape of driver mutations in melanoma. Cell.

[62] Omholt K., Platz A., Kanter L., Ringborg U., Hansson J. (2003). NRAS and BRAF mutations arise early during melanoma pathogenesis and are preserved throughout tumor progression. Clin. Cancer Res..

[63] Bastian B.C. (2014). The molecular pathology of melanoma: An integrated taxonomy of melanocytic neoplasia. Annu. Rev. Pathol..

[64] Patton E.E., Widlund H.R., Kutok J.L., Kopani K.R., Amatruda J.F., Murphey R.D., Berghmans S., Mayhall E.A., Traver D., Fletcher C.D.M. (2005). BRAF Mutations Are Sufficient to Promote Nevi Formation and Cooperate with p53 in the Genesis of Melanoma. Curr. Biol..

[65] Zhang Y., Xiong Y., Yarbrough W.G. (1998). ARF promotes MDM2 degradation and stabilizes p53: ARF-INK4a locus deletion impairs both the Rb and p53 tumor suppression pathways. Cell.

[66] Beroukhim R., Getz G., Nghiemphu L., Barretina J., Hsueh T., Linhart D., Vivanco I., Lee J.C., Huang J.H., Alexander S. (2007). Assessing the significance of chromosomal aberrations in cancer: Methodology and application to glioma. Proc. Natl. Acad. Sci. USA.

[67] Lin W.M., Baker A.C., Beroukhim R., Winckler W., Feng W., Marmion J.M., Laine E., Greulich H., Tseng H., Gates C. (2008). Modeling genomic diversity and tumor dependency in malignant melanoma. Cancer Res..

[68] Ceol C.J., Houvras Y., Jane-Valbuena J., Bilodeau S., Orlando D.A., Battisti V., Fritsch L., Lin W.M., Hollmann T.J., Ferré F. (2011). The histone methyltransferase SETDB1 is recurrently amplified in melanoma and accelerates its onset. Nature.

[69] Macgregor S., Montgomery G.W., Liu J.Z., Zhao Z.Z., Henders A.K., Stark M., Schmid H., Holland E.A., Duffy D.L., Zhang M. (2011). Genome-wide association study identifies a new melanoma susceptibility locus at 1q21.3. Nat. Genet..

[70] Lian C.G., Xu Y., Ceol C., Wu F., Larson A., Dresser K., Xu W., Tan L., Hu Y., Zhan Q. (2012). Loss of 5-hydroxymethylcytosine is an epigenetic hallmark of melanoma. Cell.

[71] Dovey M., White R.M., Zon L.I. (2009). Oncogenic NRAS cooperates with p53 loss to generate melanoma in zebrafish. Zebrafish.

[72] Michailidou C., Jones M., Walker P., Kamarashev J., Kelly A., Hurlstone A.F.L. (2009). Dissecting the roles of Raf- and PI3K-signalling pathways in melanoma formation and progression in a zebrafish model. Dis. Model. Mech..

[73] Santoriello C., Gennaro E., Anelli V., Distel M., Kelly A., Köster R.W., Hurlstone A., Mione M. (2010). Kita driven expression of oncogenic HRAS leads to early onset and highly penetrant melanoma in zebrafish. PLoS ONE.

[74] Colanesi S., Taylor K.L., Temperley N.D., Lundegaard P.R., Liu D., North T.E., Ishizaki H., Kelsh R.N., Patton E.E. (2012). Small molecule screening identifies targetable zebrafish pigmentation pathways. Pigment Cell Melanoma Res..

[75] White R.M., Cech J., Ratanasirintrawoot S., Lin C.Y., Rahl P.B., Burke C.J., Langdon E., Tomlinson M.L., Mosher J., Kaufman C. (2011). DHODH modulates transcriptional elongation in the neural crest and melanoma. Nature.

[76] Brady C.A., Rennekamp A.J., Peterson R.T. (2016). Chemical Screening in Zebrafish. Methods Mol. Biol..

[77] McLean J.E., Neidhardt E.A., Grossman T.H., Hedstrom L. (2001). Multiple inhibitor analysis of the brequinar and leflunomide binding sites on human dihydroorotate dehydrogenase. Biochemistry.

[78] Van Rooijen E., Fazio M., Zon L.I. (2017). From fish bowl to bedside: The power of zebrafish to unravel melanoma pathogenesis and discover new therapeutics. Pigment Cell Melanoma Res..

[79] Fernandez Del Ama L., Jones M., Walker P., Chapman A., Braun J.A., Mohr J., Hurlstone A.F.L. (2016). Reprofiling using a zebrafish melanoma model reveals drugs cooperating with targeted therapeutics. Oncotarget.

[80] Lee L.M.J., Seftor E.A., Bonde G., Cornell R.A., Hendrix M.J.C. (2005). The fate of human malignant melanoma cells transplanted into zebrafish embryos: Assessment of migration and cell division in the absence of tumor formation. Dev. Dyn..

[81] Nicoli S., Presta M. (2007). The zebrafish/tumor xenograft angiogenesis assay. Nat. Protoc..

[82] Li P., White R.M., Zon L.I. (2011). Transplantation in zebrafish. Methods Cell Biol..

[83] Topczewska J.M., Postovit L.-M., Margaryan N.V., Sam A., Hess A.R., Wheaton W.W., Nickoloff B.J., Topczewski J., Hendrix M.J.C. (2006). Embryonic and tumorigenic pathways converge via Nodal signaling: Role in melanoma aggressiveness. Nat. Med..

[84] Haldi M., Ton C., Seng W.L., McGrath P. (2006). Human melanoma cells transplanted into zebrafish proliferate, migrate, produce melanin, form masses and stimulate angiogenesis in zebrafish. Angiogenesis.

[85] White R.M., Sessa A., Burke C., Bowman T., LeBlanc J., Ceol C., Bourque C., Dovey M., Goessling W., Burns C.E. (2008). Transparent adult zebrafish as a tool for in vivo transplantation analysis. Cell Stem Cell.

[86] Van Der Ent W., Burrello C., Teunisse A.F.A.S., Ksander B.R., Van Der Velden P.A., Jager M.J., Jochemsen A.G., Snaar-Jagalska B.E. (2014). Modeling of human uveal melanoma in zebrafish xenograft embryos. Investig. Ophthalmol. Vis. Sci..

[87] Kansler E.R., Verma A., Langdon E.M., Simon-Vermot T., Yin A., Lee W., Attiyeh M., Elemento O., White R.M. (2017). Melanoma genome evolution across species. BMC Genom..

[88] Heilmann S., Ratnakumar K., Langdon E.M., Kansler E.R., Kim I.S., Campbell N.R., Perry E.B., McMahon A.J., Kaufman C.K., van Rooijen E. (2015). A Quantitative System for Studying Metastasis Using Transparent Zebrafish. Cancer Res..

[89] Niezgoda A., Niezgoda P., Czajkowski R. (2015). Novel Approaches to Treatment of Advanced Melanoma: A Review on Targeted Therapy and Immunotherapy. Biomed Res. Int..

[90] Ostrom Q.T., Bauchet L., Davis F.G., Deltour I., Fisher J.L., Langer C.E., Pekmezci M., Schwartzbaum J.A., Turner M.C., Walsh K.M. (2014). The epidemiology of glioma in adults: A “state of the science” review. Neuro Oncol..

[91] Louis D.N., Perry A., Reifenberger G., von Deimling A., Figarella-Branger D., Cavenee W.K., Ohgaki H., Wiestler O.D., Kleihues P., Ellison D.W. (2016). The 2016 World Health Organization Classification of Tumors of the Central Nervous System: A summary. Acta Neuropathol..

[92] Franceschi E., Minichillo S., Brandes A.A. (2017). Pharmacotherapy of Glioblastoma: Established Treatments and Emerging Concepts. CNS Drugs.

[93] Stupp R., Mason W.P., van den Bent M.J., Weller M., Fisher B., Taphoorn M.J.B., Belanger K., Brandes A.A., Marosi C., Bogdahn U. (2005). European Organisation for Research and Treatment of Cancer Brain Tumor and Radiotherapy Groups; National Cancer Institute of Canada Clinical Trials Group Radiotherapy plus concomitant and adjuvant temozolomide for glioblastoma. N. Engl. J. Med..

[94] Stavrovskaya A.A., Shushanov S.S., Rybalkina E.Y. (2016). Problems of Glioblastoma Multiforme Drug Resistance. Biochemistry.

[95] Huse J.T., Holland E.C. (2010). Targeting brain cancer: Advances in the molecular pathology of malignant glioma and medulloblastoma. Nat. Rev. Cancer.

[96] Ceccarelli M., Barthel F.P., Malta T.M., Sabedot T.S., Salama S.R., Murray B.A., Morozova O., Newton Y., Radenbaugh A., Pagnotta S.M. (2016). Molecular Profiling Reveals Biologically Discrete Subsets and Pathways of Progression in Diffuse Glioma. Cell.

[97] Lenting K., Verhaak R., Ter Laan M., Wesseling P., Leenders W. (2017). Glioma: Experimental models and reality. Acta Neuropathol..

[98] Cancer Genome Atlas Research Network (2008). Comprehensive genomic characterization defines human glioblastoma genes and core pathways. Nature.

[99] Verhaak R.G.W., Hoadley K.A., Purdom E., Wang V., Qi Y., Wilkerson M.D., Miller C.R., Ding L., Golub T., Mesirov J.P. (2010). Integrated genomic analysis identifies clinically relevant subtypes of glioblastoma characterized by abnormalities in PDGFRA, IDH1, EGFR, and NF1. Cancer Cell.

[100] Verhaak R.G.W. (2016). Moving the needle: Optimizing classification for glioma. Sci. Transl. Med..

[101] Brennan C.W., Verhaak R.G.W., McKenna A., Campos B., Noushmehr H., Salama S.R., Zheng S., Chakravarty D., Sanborn J.Z., Berman S.H. (2013). TCGA Research Network The somatic genomic landscape of glioblastoma. Cell.

[102] Guan X., Vengoechea J., Zheng S., Sloan A.E., Chen Y., Brat D.J., O’Neill B.P., de Groot J., Yust-Katz S., Yung W.-K.A. (2014). Molecular subtypes of glioblastoma are relevant to lower grade glioma. PLoS ONE.

[103] Zoncu R., Efeyan A., Sabatini D.M. (2011). mTOR: From growth signal integration to cancer, diabetes and ageing. Nat. Rev. Mol. Cell Biol..

[104] Viotti J., Duplan E., Caillava C., Condat J., Goiran T., Giordano C., Marie Y., Idbaih A., Delattre J.-Y., Honnorat J. (2014). Glioma tumor grade correlates with parkin depletion in mutant p53-linked tumors and results from loss of function of p53 transcriptional activity. Oncogene.

[105] Speidel D. (2015). The role of DNA damage responses in p53 biology. Arch. Toxicol..

[106] Ohgaki H., Kleihues P. (2007). Genetic pathways to primary and secondary glioblastoma. Am. J. Pathol..

[107] Nasser M.M., Mehdipour P. (2017). Exploration of Involved Key Genes and Signaling Diversity in Brain Tumors. Cell. Mol. Neurobiol..

[108] Venkatesan S., Lamfers M.L.M., Dirven C.M.F., Leenstra S. (2016). Genetic biomarkers of drug response for small-molecule therapeutics targeting the RTK/Ras/PI3K, p53 or Rb pathway in glioblastoma. CNS Oncol..

[109] Mao H., Lebrun D.G., Yang J., Zhu V.F., Li M. (2012). Deregulated signaling pathways in glioblastoma multiforme: Molecular mechanisms and therapeutic targets. Cancer Investig..

[110] Solomon D.A., Kim J.-S., Jean W., Waldman T. (2008). Conspirators in a capital crime: Co-deletion of p18INK4c and p16INK4a/p14ARF/p15INK4b in glioblastoma multiforme. Cancer Res..

[111] Shin J., Padmanabhan A., de Groh E.D., Lee J.-S., Haidar S., Dahlberg S., Guo F., He S., Wolman M.A., Granato M. (2012). Zebrafish neurofibromatosis type 1 genes have redundant functions in tumorigenesis and embryonic development. Dis. Model. Mech..

[112] Jung I.H., Leem G.L., Jung D.E., Kim M.H., Kim E.Y., Kim S.H., Park H.-C., Park S.W. (2013). Glioma is formed by active Akt1 alone and promoted by active Rac1 in transgenic zebrafish. Neuro Oncol..

[113] Ju B., Chen W., Spitsbergen J.M., Lu J., Vogel P., Peters J.L., Wang Y.-D., Orr B.A., Wu J., Henson H.E. (2014). Activation of Sonic hedgehog signaling in neural progenitor cells promotes glioma development in the zebrafish optic pathway. Oncogenesis.

[114] Ju B., Chen W., Orr B.A., Spitsbergen J.M., Jia S., Eden C.J., Henson H.E., Taylor M.R. (2015). Oncogenic KRAS promotes malignant brain tumors in zebrafish. Mol. Cancer.

[115] Mayrhofer M., Gourain V., Reischl M., Affaticati P., Jenett A., Joly J.-S., Benelli M., Demichelis F., Poliani P.L., Sieger D. (2017). A novel brain tumour model in zebrafish reveals the role of YAP activation in MAPK- and PI3K-induced malignant growth. Dis. Model. Mech..

[116] Mione M.C., Trede N.S. (2010). The zebrafish as a model for cancer. Dis. Model. Mech..

[117] White R., Rose K., Zon L. (2013). Zebrafish cancer: The state of the art and the path forward. Nat. Rev. Cancer.

[118] Fleming A., Diekmann H., Goldsmith P. (2013). Functional characterisation of the maturation of the blood-brain barrier in larval zebrafish. PLoS ONE.

[119] Eden C.J., Ju B., Murugesan M., Phoenix T.N., Nimmervoll B., Tong Y., Ellison D.W., Finkelstein D., Wright K., Boulos N. (2015). Orthotopic models of pediatric brain tumors in zebrafish. Oncogene.

[120] Tobia C., Gariano G., De Sena G., Presta M. (2013). Zebrafish embryo as a tool to study tumor/endothelial cell cross-talk. Biochim. Biophys. Acta.

[121] Lally B.E., Geiger G.A., Kridel S., Arcury-Quandt A.E., Robbins M.E., Kock N.D., Wheeler K., Peddi P., Georgakilas A., Kao G.D. (2007). Identification and biological evaluation of a novel and potent small molecule radiation sensitizer via an unbiased screen of a chemical library. Cancer Res..

[122] Geiger G.A., Fu W., Kao G.D. (2008). Temozolomide-mediated radiosensitization of human glioma cells in a zebrafish embryonic system. Cancer Res..

[123] Yang X.-J., Cui W., Gu A., Xu C., Yu S.-C., Li T.-T., Cui Y.-H., Zhang X., Bian X.-W. (2013). A novel zebrafish xenotransplantation model for study of glioma stem cell invasion. PLoS ONE.

[124] Lal S., La Du J., Tanguay R.L., Greenwood J.A. (2012). Calpain 2 is required for the invasion of glioblastoma cells in the zebrafish brain microenvironment. J. Neurosci. Res..

[125] Rampazzo E., Persano L., Pistollato F., Moro E., Frasson C., Porazzi P., Della Puppa A., Bresolin S., Battilana G., Indraccolo S. (2013). Wnt activation promotes neuronal differentiation of glioblastoma. Cell Death Dis..

[126] Welker A.M., Jaros B.D., Puduvalli V.K., Imitola J., Kaur B., Beattie C.E. (2016). Correction: Standardized orthotopic xenografts in zebrafish reveal glioma cell-line-specific characteristics and tumor cell heterogeneity. Dis. Model. Mech..

[127] Welker A.M., Jaros B.D., An M., Beattie C.E. (2017). Changes in tumor cell heterogeneity after chemotherapy treatment in a xenograft model of glioblastoma. Neuroscience.

[128] Hamilton L., Astell K.R., Velikova G., Sieger D. (2016). A Zebrafish live imaging model reveals differential responses of microglia toward glioblastoma cells in vivo. Zebrafish.

[129] Wenger A., Larsson S., Danielsson A., Elbæk K.J., Kettunen P., Tisell M., Sabel M., Lannering B., Nordborg C., Schepke E. (2017). Stem cell cultures derived from pediatric brain tumors accurately model the originating tumors. Oncotarget.

[130] Breznik B., Motaln H., Vittori M., Rotter A., Lah Turnšek T. (2017). Mesenchymal stem cells differentially affect the invasion of distinct glioblastoma cell lines. Oncotarget.

[131] Vittori M., Breznik B., Gredar T., Hrovat K., Bizjak Mali L., Lah T.T. (2016). Imaging of human glioblastoma cells and their interactions with mesenchymal stem cells in the zebrafish (*Danio rerio*) embryonic brain. Radiol. Oncol..

[132] Lawson N.D., Weinstein B.M. (2002). In vivo imaging of embryonic vascular development using transgenic zebrafish. Dev. Biol..

[133] Yang X., Cui W., Yu S., Xu C., Chen G., Gu A., Li T., Cui Y., Zhang X., Bian X. (2014). A synthetic dl-nordihydroguaiaretic acid (Nordy), inhibits angiogenesis, invasion and proliferation of glioma stem cells within a zebrafish xenotransplantation model. PLoS ONE.

[134] Hernan R., Fasheh R., Calabrese C., Frank A.J., Maclean K.H., Allard D., Barraclough R., Gilbertson R.J. (2003). ERBB2 up-regulates S100A4 and several other prometastatic genes in medulloblastoma. Cancer Res..

[135] Modzelewska K., Boer E.F., Mosbruger T.L., Picard D., Anderson D., Miles R.R., Kroll M., Oslund W., Pysher T.J., Schiffman J.D. (2016). MEK inhibitors reverse growth of embryonal brain tumors derived from oligoneural precursor cells. Cell Rep..

[136] Lister J.A., Robertson C.P., Lepage T., Johnson S.L., Raible D.W. (1999). nacre encodes a zebrafish microphthalmia-related protein that regulates neural-crest-derived pigment cell fate. Development.

[137] Schwartzbaum J.A., Fisher J.L., Aldape K.D., Wrensch M. (2006). Epidemiology and molecular pathology of glioma. Nat. Clin. Pract. Neurol..

[138] Löhr H., Hammerschmidt M. (2011). Zebrafish in endocrine systems: Recent advances and implications for human disease. Annu. Rev. Physiol..

[139] Liu N.-A., Jiang H., Ben-Shlomo A., Wawrowsky K., Fan X.-M., Lin S., Melmed S. (2011). Targeting zebrafish and murine pituitary corticotroph tumors with a cyclin-dependent kinase (CDK) inhibitor. Proc. Natl. Acad. Sci. USA.

[140] Cirello V., Gaudenzi G., Grassi E.S., Colombo C., Vicentini L., Ferrero S., Persani L., Vitale G., Fugazzola L. (2017). Tumor and normal thyroid spheroids: From tissues to zebrafish. Minerva Endocrinol..

[141] Gaudenzi G., Albertelli M., Dicitore A., Würth R., Gatto F., Barbieri F., Cotelli F., Florio T., Ferone D., Persani L. (2017). Patient-derived xenograft in zebrafish embryos: A new platform for translational research in neuroendocrine tumors. Endocrine.

